# Supplementary material for: Auxiliary sequential deposition enables 19%-efficiency organic solar cells processed from halogen-free solvents
Source: Nat Commun. 2023 Oct 31;14:6964. doi: 10.1038/s41467-023-41978-0 (PMC10618449; doi:10.1038/s41467-023-41978-0)
Supplement: Supplementary file 1 — Supplementary Information [file 41467_2023_41978_MOESM1_ESM.pdf]

Supplementary Information

Auxiliary sequential deposition enables 19%-Efficiency Organic Solar Cells processed from Halogen-free Solvents

Siwei Luo, Chao Li, Jianquan Zhang, Xinhui Zou, Heng Zhao, Kan Ding, Hui Huang, Jiali Song, Jicheng Yi, Han Yu, Kam Sing Wong, Guangye Zhang, Ade Harald, Wei Ma, Huawei Hu, Yanming Sun,* He Yan*

Electrochemical and photophysical characterization

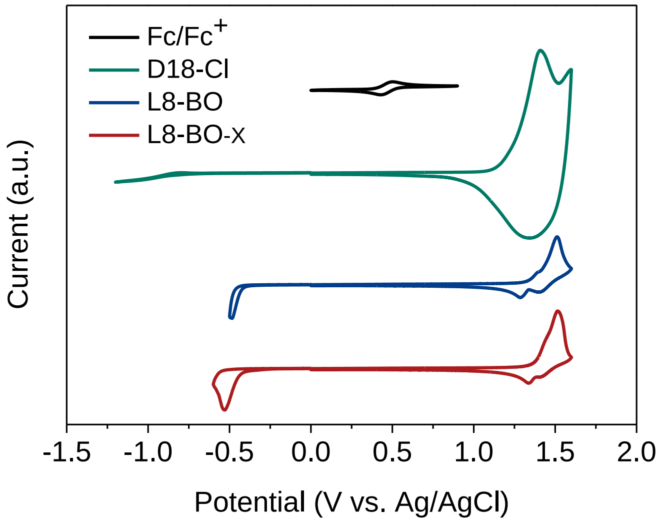

Supplementary Figure 1. CV curves of and D18-Cl, L8-BO, and L8-BO-X.

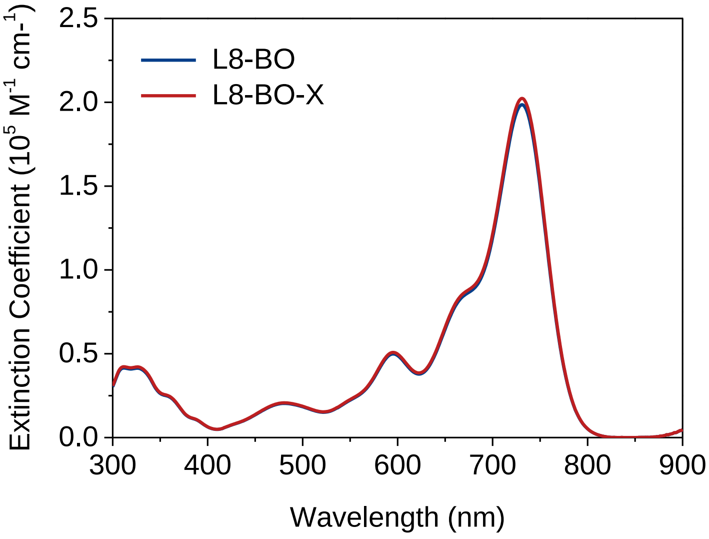

Supplementary Figure 2. Absorption spectra of the NFAs in 5×10^{-6} M chloroform solution.

Supplementary Table 1. Summary of the optical and electrochemical properties of D18-Cl, L8-BO and L8-BO-X.

	HOMO/LUMO (eV)	Abs. peak (nm)	Abs. edge (nm)	Optical bandgap (eV)
D18-Cl		581	627	1.98
L8-BO	-5.75/-3.91	796	887	1.40
L8-BO-X	-5.74/-3.89	806	892	1.39

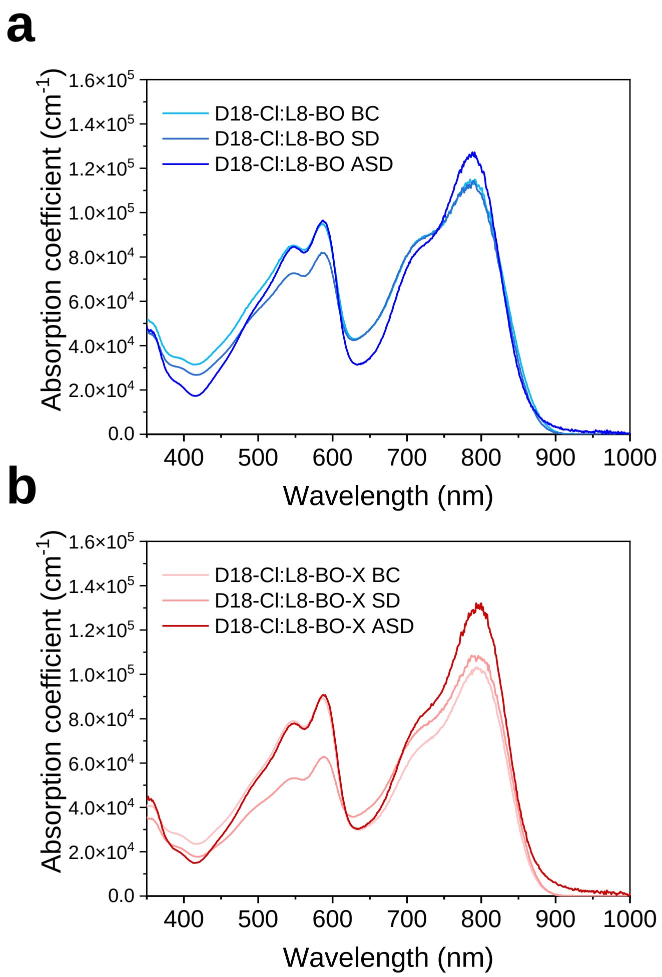

Supplementary Figure 3. Absorption spectra of the blend films for (a) D18-Cl:L8-BO and (b) D18-Cl:L8-BO-X from the BC, SD and ASD methods.

Device optimization

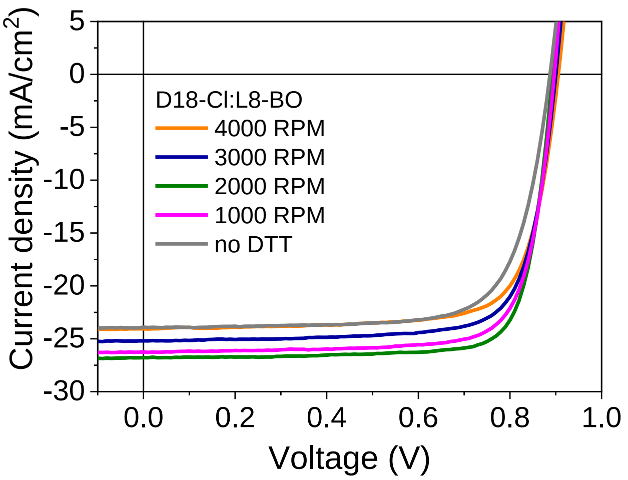

Supplementary Figure 4. *J-V* curves of D18-Cl:L8-BO with different DTT treatments.

Supplementary Table 2. Device data of D18-Cl:L8-BO with different DTT treatments.

D18-Cl:L8-BO	V_{OC} (V)	J_{SC} (mA cm^{-2})	FF (%)	PCE (%)
No DTT	0.881	23.09	76.2	15.50
4000 rpm	0.916	24.60	75.3	16.98
3000 rpm	0.911	25.29	75.7	17.43
2000 rpm	0.896	26.37	76.3	18.03
1000 rpm	0.907	25.86	76.0	17.82

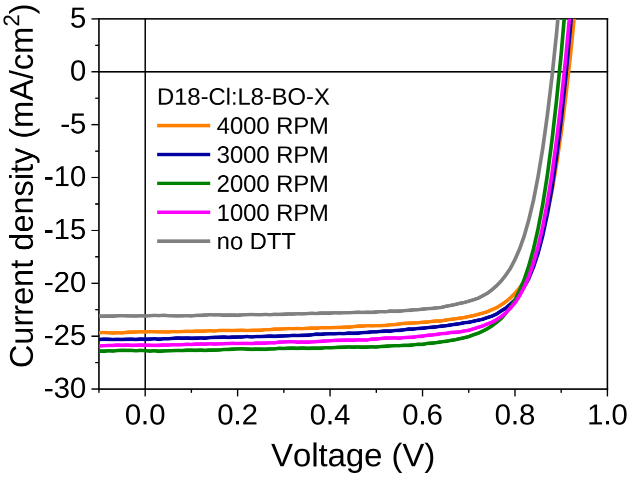

Supplementary Figure 5. *J-V* curves of D18-Cl:L8-BO-X with different DTT treatments.

Supplementary Table 3. Device data of D18-Cl:L8-BO-X with different DTT treatments.

D18-Cl:L8-BO-X	V_{OC} (V)	J_{SC} (mA cm^{-2})	FF (%)	PCE (%)
No DTT	0.887	23.91	74.1	15.73
4000 rpm	0.906	24.66	75.5	16.85
3000 rpm	0.900	25.19	76.5	17.34
2000 rpm	0.893	26.78	79.6	19.04
1000 rpm	0.897	26.25	77.5	18.24

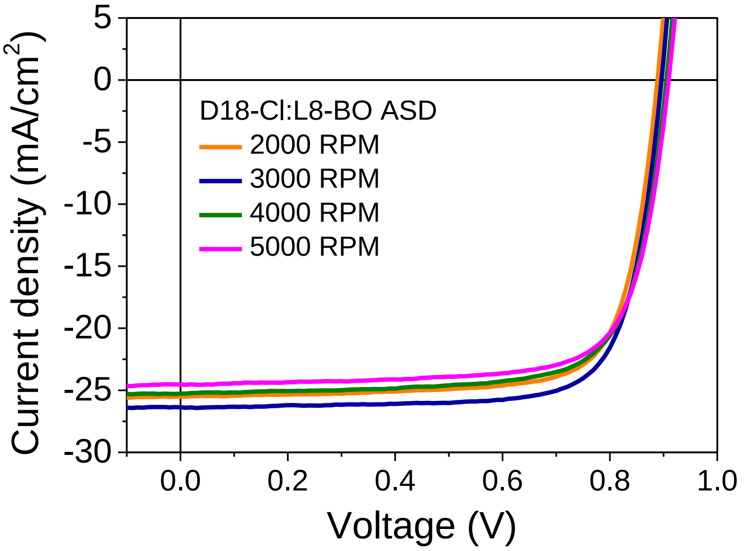

Supplementary Figure 6. *J-V* curves of D18-Cl:L8-BO with different spin-coating rate of L8-BO.

Supplementary Table 4. Device data of the ASD devices of D18-Cl:L8-BO with different spin-coating rate of L8-BO.

L8-BO	V_{OC} (V)	J_{SC} (mA cm^{-2})	FF (%)	PCE (%)
5000 rpm	0.910	24.53	74.7	16.65
4000 rpm	0.905	25.27	74.2	16.97
3000 rpm	0.896	26.37	76.3	18.03
2000 rpm	0.889	25.52	75.8	17.20

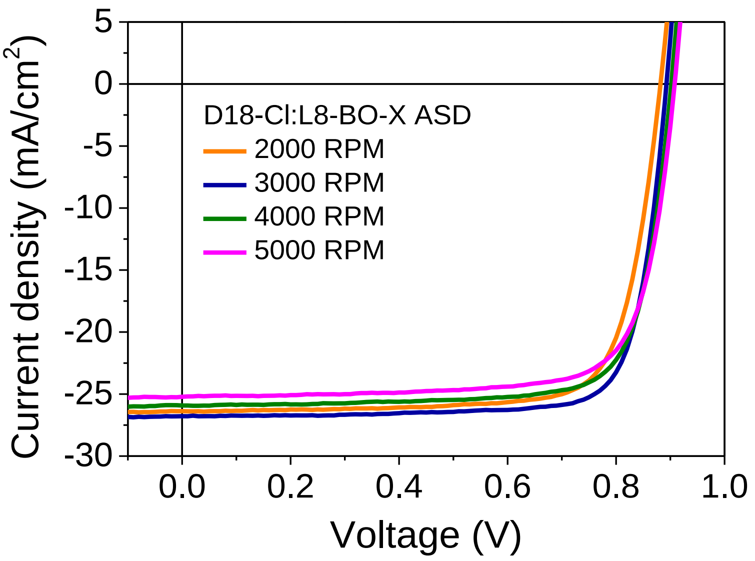

Supplementary Figure 7. *J-V* curves of D18-Cl:L8-BO-X with different spin-coating rate of L8-BO-X.

Supplementary Table 5. Device data of the ASD devices of D18-Cl:L8-BO-X with different spin-coating rate of L8-BO-X.

L8-BO-X	V_{OC} (V)	J_{SC} (mA cm^{-2})	FF (%)	PCE (%)
5000 rpm	0.908	25.22	76.1	17.42
4000 rpm	0.902	25.92	77.6	18.14
3000 rpm	0.893	26.78	79.6	19.04
2000 rpm	0.882	26.38	77.1	17.93

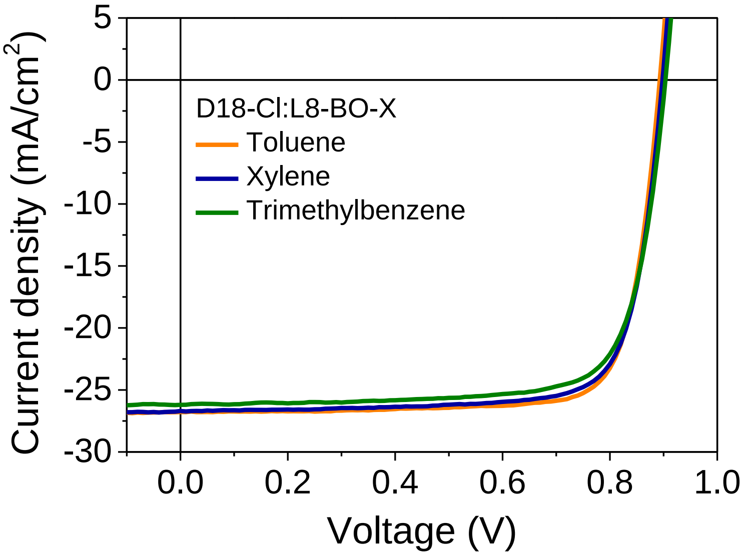

Supplementary Figure 8. *J-V* curves of the ASD devices of D18-Cl:L8-BO-X with different halogen-free solvents.

Supplementary Table 6. Device data of the ASD devices of D18-Cl:L8-BO-X with different halogen-free solvents.

L8-BO-X	V_{OC} (V)	J_{SC} (mA cm^{-2})	FF (%)	PCE (%)
Toluene	0.893	26.78	79.6	19.04
Xylene	0.898	26.70	78.0	18.69
TMB	0.903	26.20	76.5	18.10

Stability test.

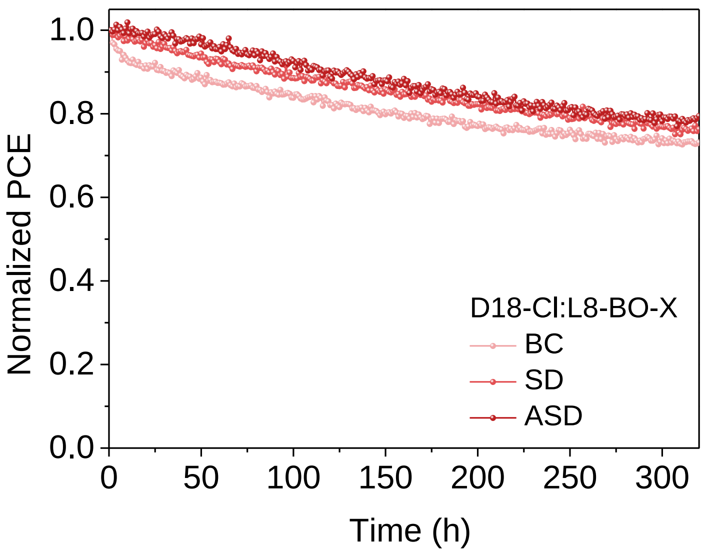

Supplementary Figure 9. Photostability of D18-Cl:L8-BO-X under the MPP tracking mode.

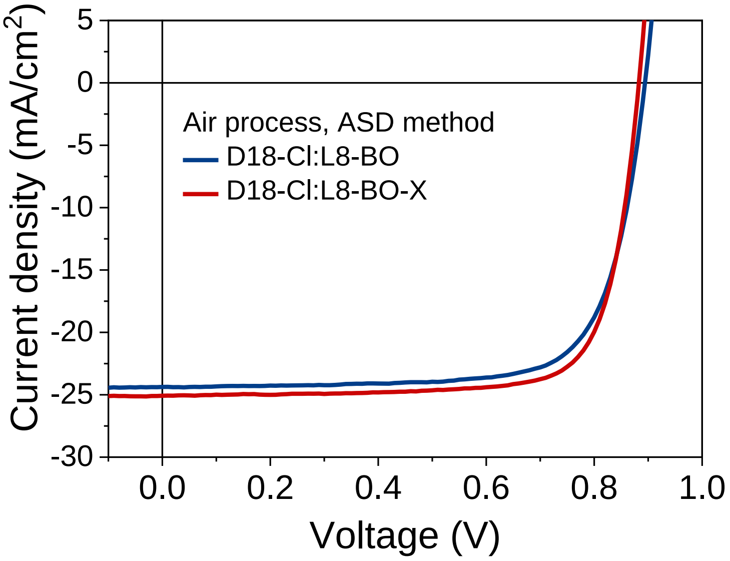

Supplementary Figure 10. *J-V* curves of the ASD devices of D18-Cl:L8-BO and D18-Cl:L8-BO-X under ambient conditions.

Supplementary Table 7. Device data of the ASD devices of D18-Cl:L8-BO and D18-Cl:L8-BO-X under ambient conditions.

	V_{OC} (V)	J_{SC} (mA cm^{-2})	FF (%)	PCE (%)
D18-Cl:L8-BO	0.894	25.31	74.43	16.84
D18-Cl:L8-BO-X	0.883	25.08	77.09	17.07

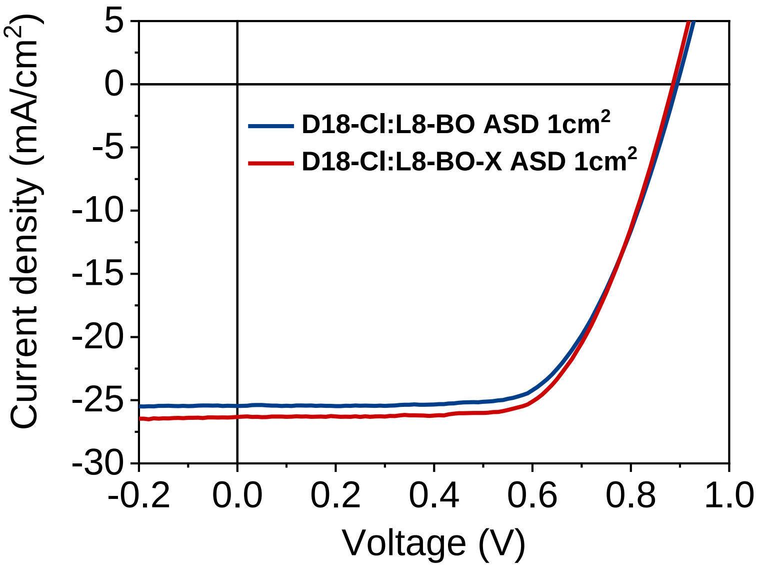

Supplementary Figure 11. *J-V* curves of the ASD 1cm^2 area devices of D18-Cl:L8-BO and D18-Cl:L8-BO-X.

Supplementary Table 8. Large area devices data of the ASD devices of D18-Cl:L8-BO and D18-Cl:L8-BO-X.

	V_{OC} (V)	J_{SC} (mA cm^{-2})	FF (%)	PCE (%)
D18-Cl:L8-BO 1cm^2	0.894	25.461	0.646	14.696
D18-Cl:L8-BO-X 1cm^2	0.886	26.328	0.653	15.227

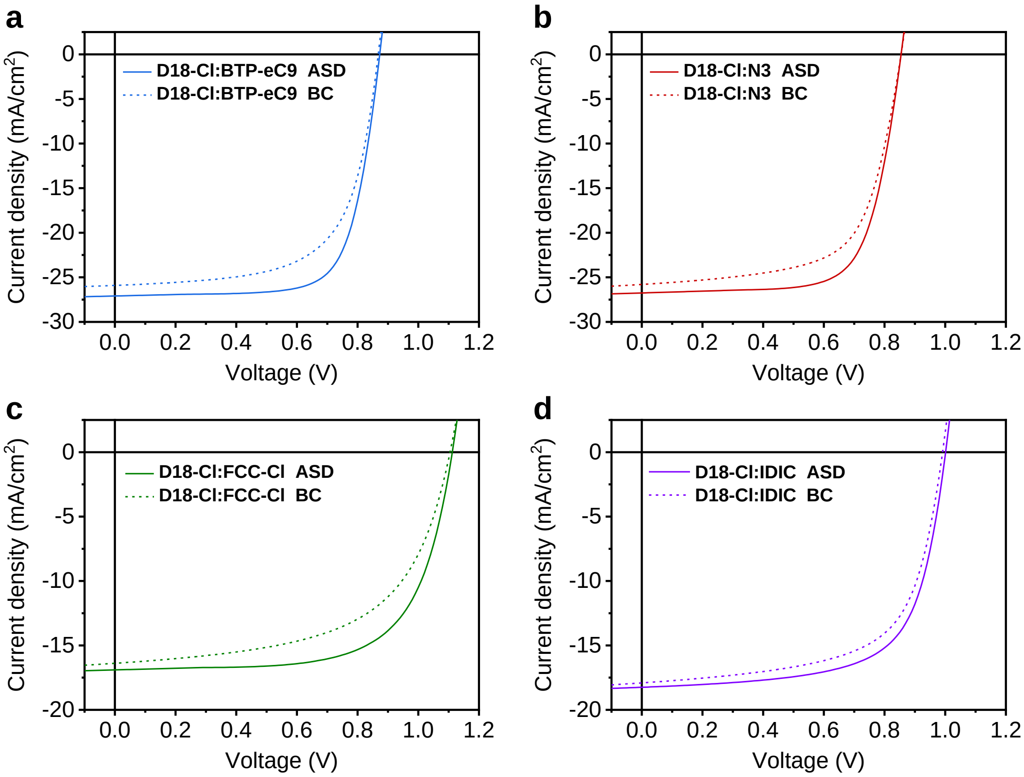

Supplementary Figure 12. Universality test of ASD method based on multiple acceptors of (a) BTP-eC9, (b) N3, (c) FCC-Cl, and (d) IDIC.

Supplementary Table 9. Universality test data of the ASD devices based on multiple acceptors of BTP-eC9, N3, FCC-Cl, and IDIC.

	V_{OC} (V)	J_{SC} (mA cm^{-2})	FF (%)	PCE (%)
D18-Cl:BTP-eC9 ASD	0.873	26.883	0.733	17.195
D18-Cl:BTP-eC9 BC	0.868	25.909	0.647	14.545
D18-Cl:N3 ASD	0.855	26.213	0.721	16.140
D18-Cl: N3 BC	0.854	25.802	0.646	14.251
D18-Cl:FCC-Cl ASD	1.112	16.635	0.678	12.546
D18-Cl: FCC-Cl BC	1.105	16.386	0.575	10.400
D18-Cl:IDIC ASD	1.001	18.251	0.665	12.148
D18-Cl: IDIC BC	0.991	17.909	0.634	11.254

SCLC Mobility measurement

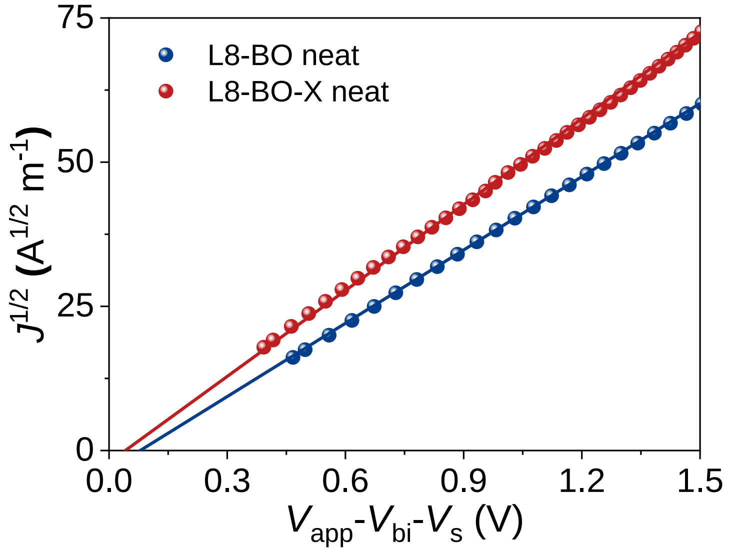

Supplementary Figure 13. SCLC mobility measurements of the neat L8-BO and L8-BO-X films (electron-only devices).

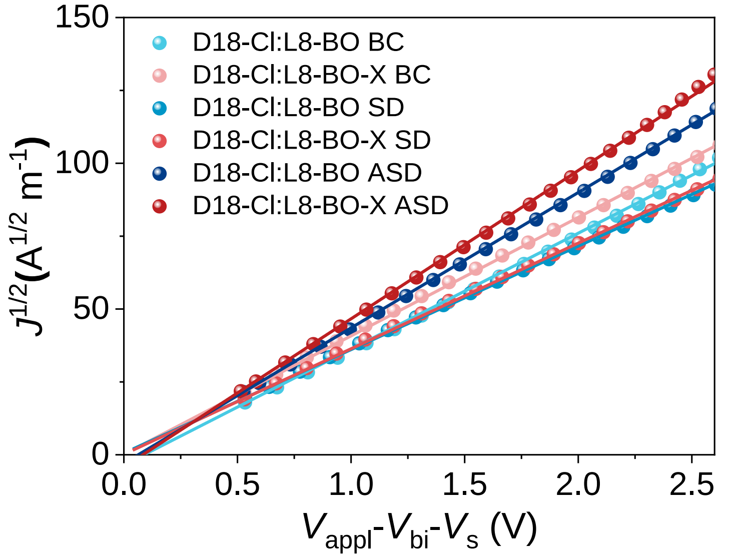

Supplementary Figure 14. SCLC mobility measurements of the D18-Cl:L8-BO and D18-Cl:L8-BO-X films processed from the BC, SD, and ASD methods (electron-only devices).

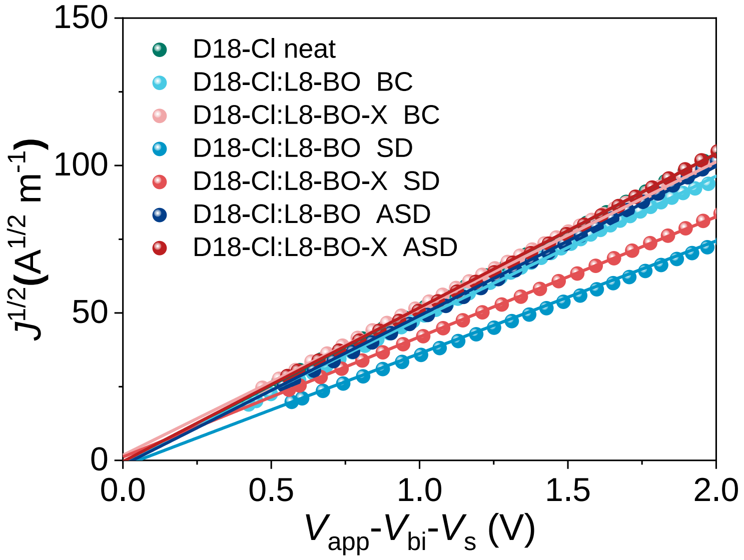

Supplementary Figure 15. SCLC mobility measurements of the neat D18-Cl, and the D18-Cl:L8-BO and D18-Cl:L8-BO-X films processed from the BC, SD, and ASD methods (hole-only devices).

Supplementary Table 10. Summary of SCLC mobility data.

	Electron mobility (cm^2 V^{-1} s^{-1})	Hole mobility (cm^2 V^{-1} s^{-1})	μ_h/μ_e
L8-BO	6.09×10^{-4}		
L8-BO-X	8.23×10^{-4}		
D18-Cl		9.32×10^{-4}	
D18-Cl:L8-BO BC	5.29×10^{-4}	7.87×10^{-4}	1.49
D18-Cl:L8-BO-X BC	5.38×10^{-4}	7.92×10^{-4}	1.47
D18-Cl:L8-BO SD	4.10×10^{-4}	5.12×10^{-4}	1.24
D18-Cl:L8-BO-X SD	4.28×10^{-4}	5.53×10^{-4}	1.29
D18-Cl:L8-BO ASD	7.37×10^{-4}	8.69×10^{-4}	1.18
D18-Cl:L8-BO-X ASD	8.88×10^{-4}	9.28×10^{-4}	1.05

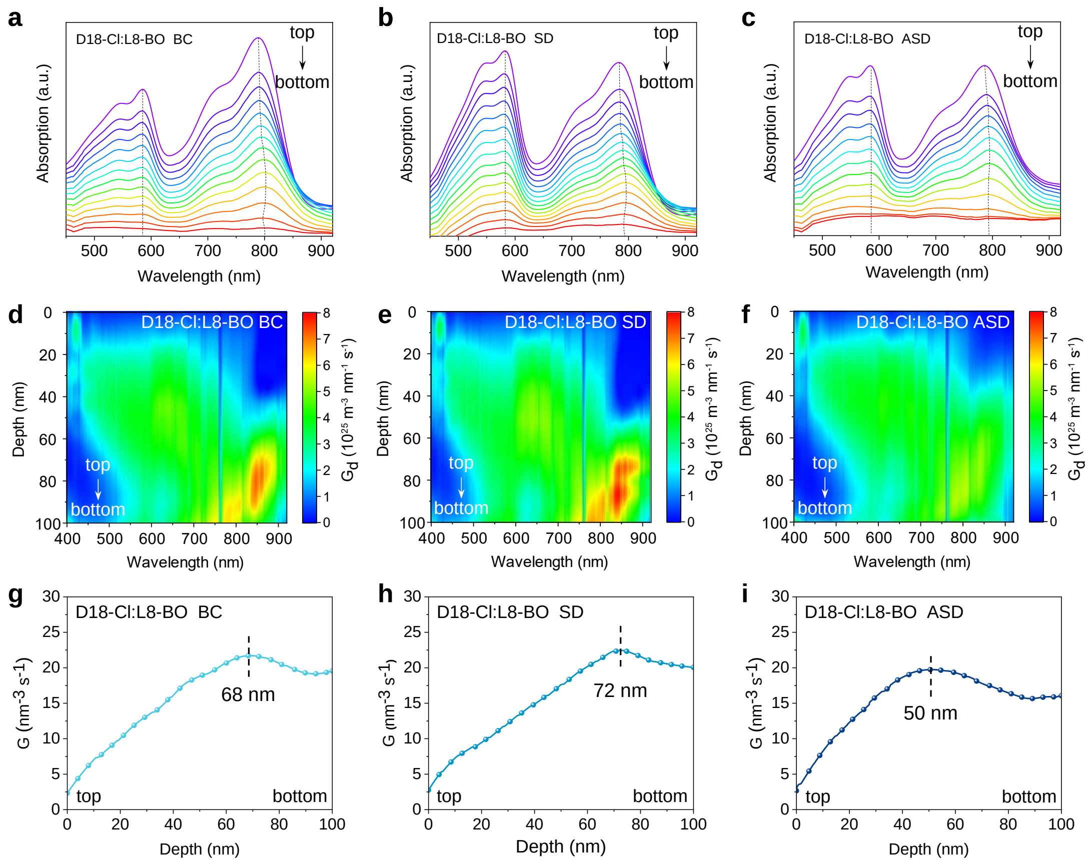

Supplementary Figure 16. The FLAS characteristics of D18-Cl:L8-BO from (a) BC, (b) SD, and (c) ASD processing. Numerical simulations for the exciton generation contours of D18-Cl:L8-BO from (d) BC, (e) ASD, and (f) ASD processing, where the noise arise from the fluctuation of the AM 1.5G solar spectrum. Dependence of the simulated exciton generation rate (G) on the film depth of D18-Cl:L8-BO from (g) BC, (h) SD, and (i) ASD processing.

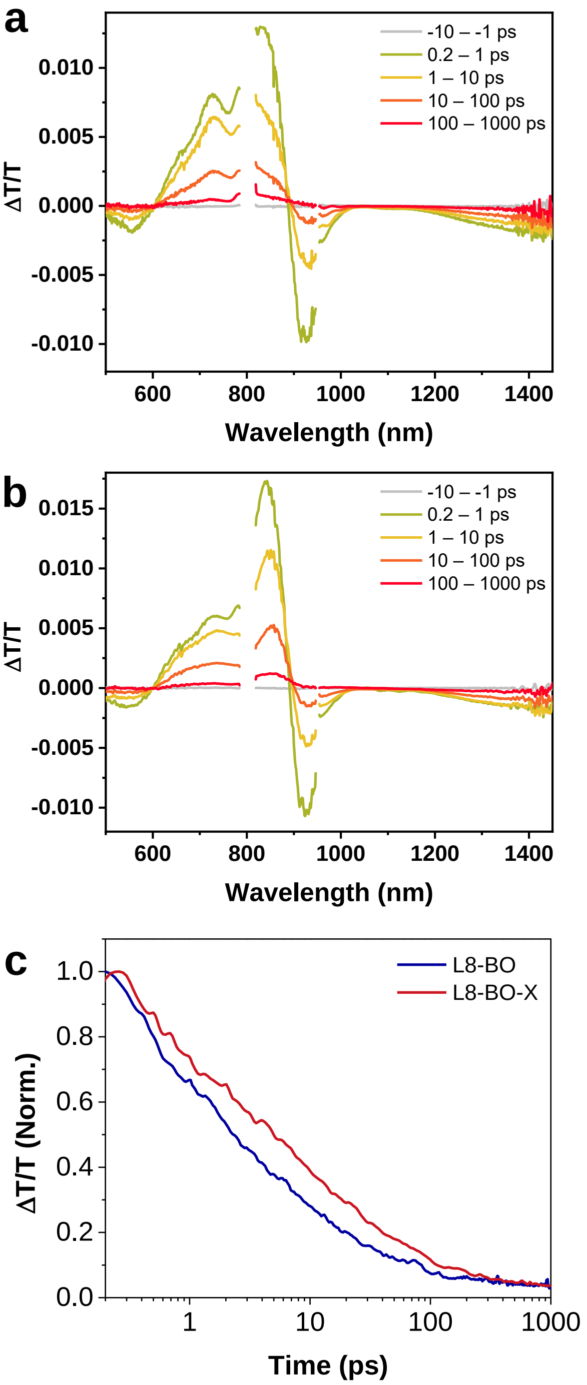

Supplementary Figure 17. TAS profiles of (a) pure L8-BO and (b) pure L8-BO-X, and (c) the corresponding TA decay.

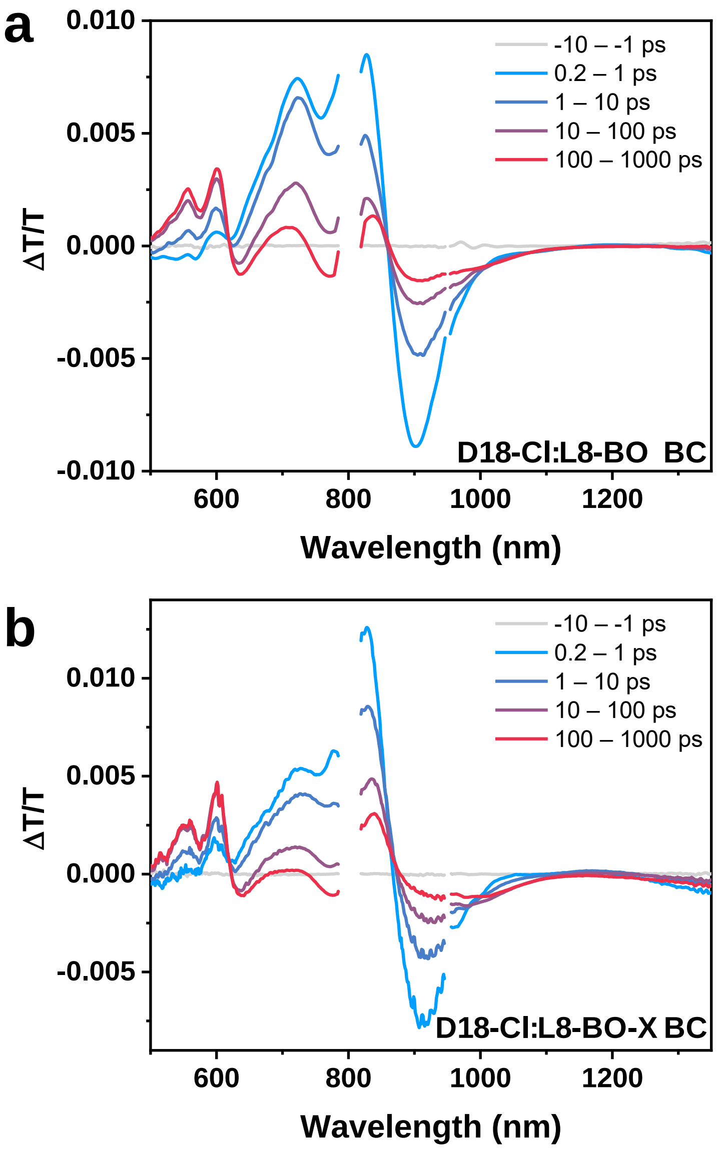

Supplementary Figure 18. TAS profiles of the (a) D18-Cl:L8-BO and (b) D18-Cl:L8-BO-X devices processed from the BC method.

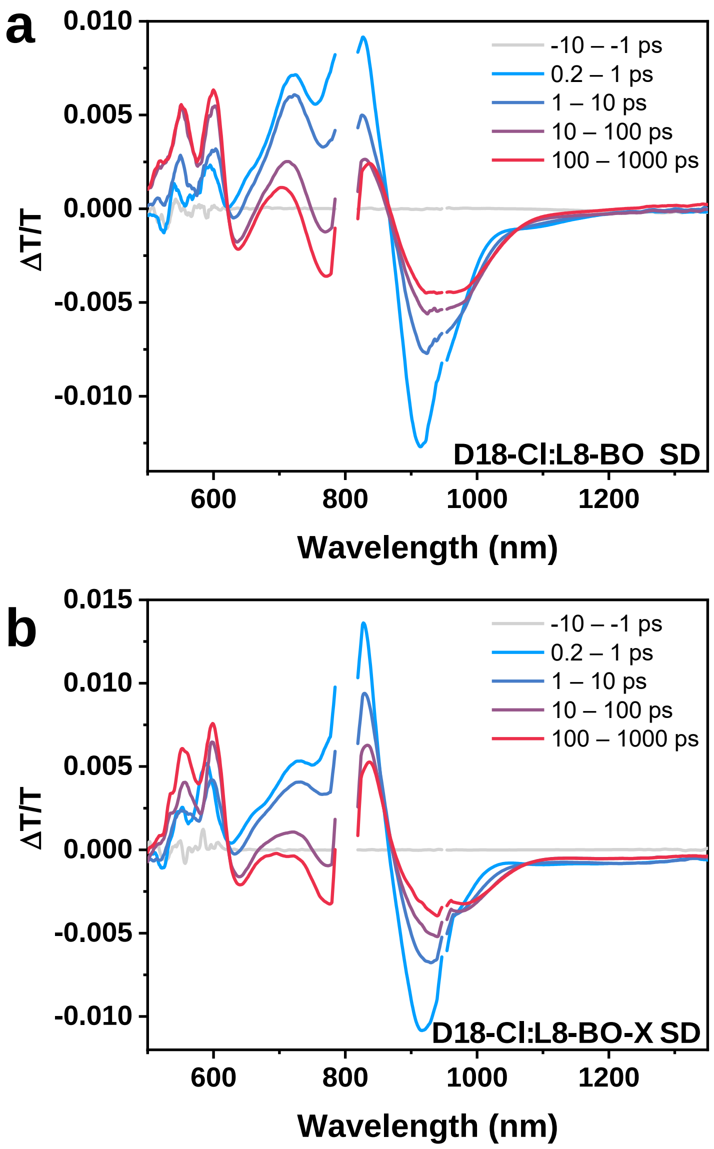

Supplementary Figure 19. TAS profiles of the (a) D18-Cl:L8-BO and (b) D18-Cl:L8-BO-X devices processed from the SD method.

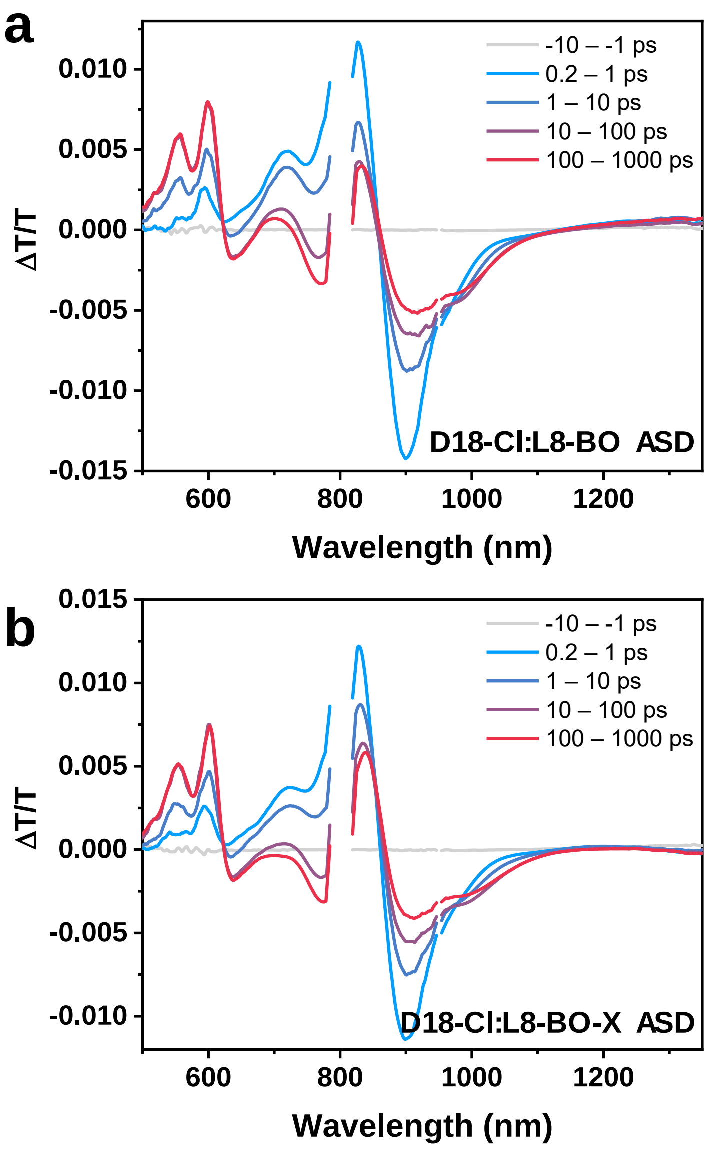

Supplementary Figure 20. TAS profiles of the (a) D18-Cl:L8-BO and (b) D18-Cl:L8-BO-X devices processed from the ASD method.

Supplementary Table 11. Lifetime data fitting from the TAS profiles.

	processing method	τ_1 (ps)	τ_2 (ps)
D18-Cl:L8-BO	BC	1.12	20.59
	SD	2.37	43.93
	ASD	0.54	12.76
D18-Cl:L8-BO-X	BC	0.61	16.01
	SD	3.76	29.32
	ASD	0.54	10.97

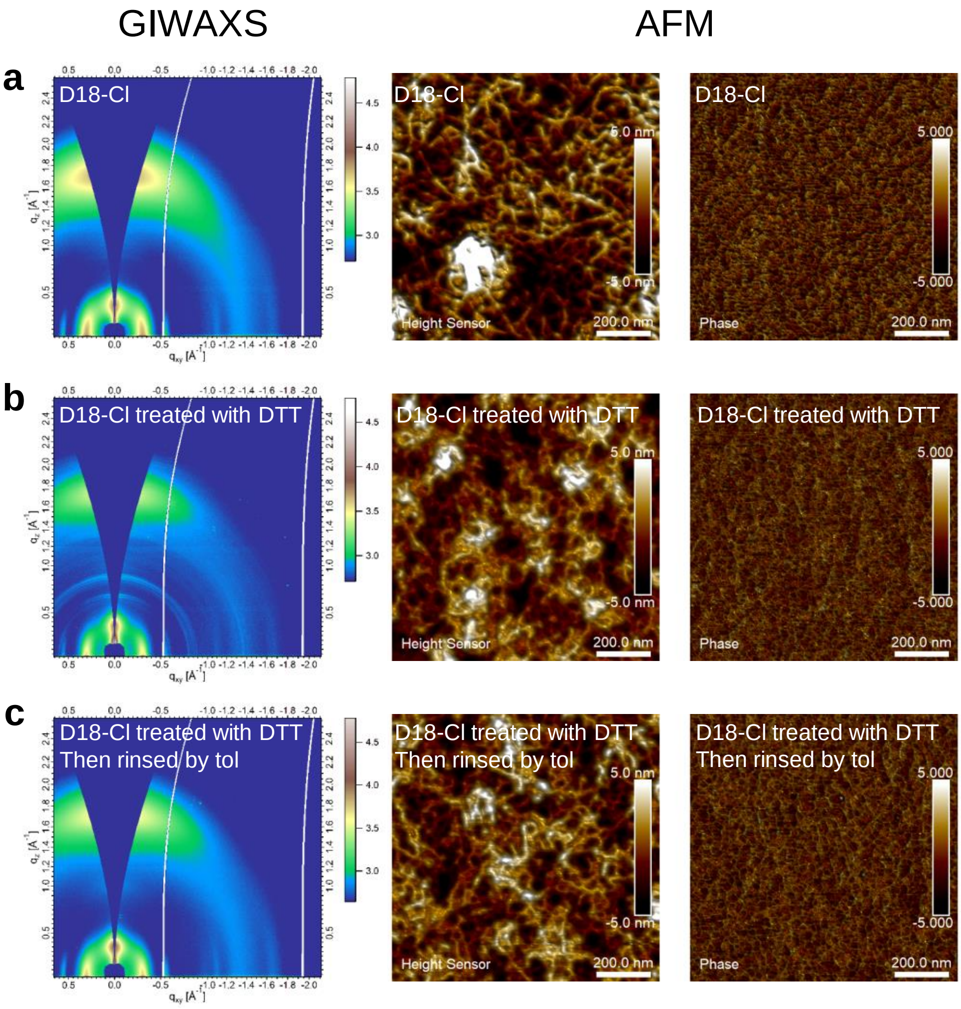

Supplementary Figure 21. GIWAXS and AFM of (a) D18-Cl, (b) D18-Cl treated by spin-coating DDT solution on top, and (c) D18-Cl treated by spin-coating DDT solution and then toluene for rinse.

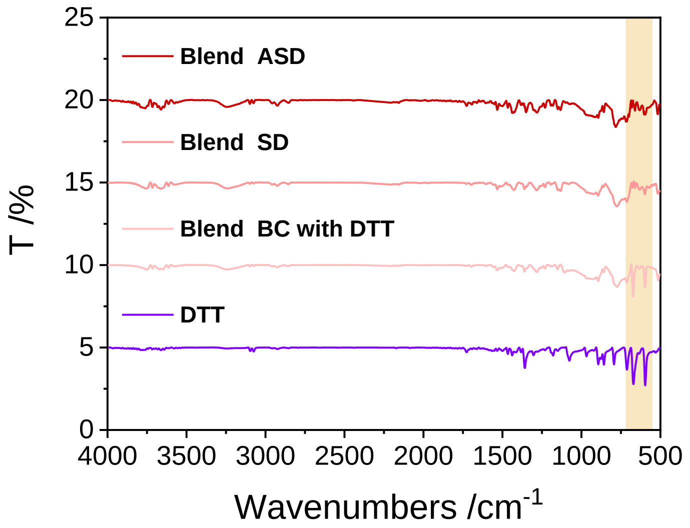

Supplementary Figure 22. FTIR spectrum of DTT and D18-Cl:L8-BO-X blend films from BC (100 wt% DTT in CF and low temperature BC), SD and ASD methods.

Supplementary Table 12. The molecular weight and PDI details of D18-Cl.

Sample Name	Mp	Mn	Mw	Mv	Mz	Mz + 1	PD
**D18-Cl**	129903	52478	142214	249434	268913	414120	2.71

Supplementary Table 13. Representative halogen-free processed OSC devices from literature.

Materials	solvent	additive	V_{OC} (V)	J_{SC} (mA cm^{-2})	FF (%)	PCE (%)	ref
PM6:5BDTBDD:BTP-BO-4Cl	o-XY	DIO	0.84	26.8	77.4	17.5	1
PM6:5BDDBDT:BTP-BO-4Cl	o-XY	DIO	0.84	26.7	77.4	17.3	1
PM6:Y6	o-XY:CS2		0.84	25.6	76.8	16.5	2
PM6:BO-4Cl	o-XY		0.84	26.7	79.0	17.7	3
PM6:BO-4Cl:Y6-1O	o-XY		0.85	26.8	80.0	18.3	3
PM6:BO-4Cl:Y6-1O	p-XY		0.84	27.1	80.0	18.2	3
PM6:BO-4Cl:Y6-1O	Tol		0.85	27.2	79.0	18.1	3
PM6:Y6	o-XY		0.78	22.8	65.8	11.6	3
PM6:Y6	o-XY		0.80	25.0	74.8	15.1	4
PM6:BTP-eC9	o-XY		0.84	26.8	76.3	17.2	4
PM6: PTer-N25	o-XY	2-MN	0.94	19.5	65.1	11.9	5
PM6/BO-4F	o-XY/THF	DIO	0.82	26.2	47.3	16.0	6
PBDB-T-b-PYT	THF	CN	0.89	21.0	66.1	12.4	7
PBDB-T-b-PYT	Tol	CN	0.89	20.6	68.4	12.5	8
PBDB-T-b-PYT	o-XY	CN	0.90	20.6	68.5	12.6	8
PTB7-Th:PC71BM	o-XY	EHB	0.80	17.2	67.5	9.3	9
PM6:Y6:BTO	PX	CN	0.85	26.3	74.3	16.6	10
PM6:Y6:BTO:PC71BM	PX	CN	0.85	27.1	75.8	17.4	10
PM6:A-2ThCl:A- 4Cl:PC71BM	Tol	DIO	0.86	26.3	77.0	17.4	11
PTzBI- dF:CH1007:PC71BM	o-XY:TMB		0.82	28.2	77.8	18.0	12
PM6:Y6	o-XY	DMN	0.81	23.7	68.3	13.2	13
PM6:Y6	o-XY	DMN	0.83	25.0	74.6	15.5	13
PPDT2FBT:PC61BM	o-XY		0.80	15.6	72.0	9.2	14
PPDT2FBT:PC61BM	o-XY	AA	0.80	15.6	72.0	9.2	14
PPDT2FBT:PC61BM	o-XY	DIO	0.80	11.8	66.0	6.6	14
PM6:YSe-C6	o-XY		0.85	25.9	73.0	16.1	15
PM6:PYT	o-XY	CN	0.96	10.7	53.0	5.4	16
PM6:PY2F-T	o-XY		0.86	23.5	64.8	13.1	16
PM6:BTP-BO-4Cl	Tol	BV	0.85	26.10	77.7	17.33	17
PM6:DTY6	o-XY		0.86	24.94	75.5	16.1	18
PM6:Y6	o-XY		0.81	26.6	70.3	15.6	19
PM6:Y6	TMB		0.80	26.4	70.9	15.4	19
TPD-1:IT-4F	o-XY	DIO	0.81	19.4	74.7	11.7	20
TPD-2:IT-4F	o-XY	DIO	0.81	19.6	74.2	11.8	20
TPD-3:IT-4F	o-XY	DIO	0.80	20.1	75.3	12.1	20
TPD-3F:IT-4F	o-XY	DIO	0.91	20.5	73.8	13.8	20
PM6:IT-4F	o-XY	P2	0.83	19.2	73.9	11.8	21
PM6:PC71BM	o-XY	P2	0.81	20.3	67.0	11.0	21
PTB7-Th:F10IC2	o-XY		0.76	20.3	66.8	10.7	22

PTB7-Th:F10IC2	o-XY		0.75	22.3	65.0	11.4	22
PM7:IT-4Cl	CS2		0.89	19.1	74.8	12.5	23
PM7:IT-4Cl	o-XY		0.88	20.6	62.4	10.8	23
PM7:IT-4F	CS2		0.93	17.1	73.4	11.7	23
PMT50:Y6(BO)	TMB	CN	0.84	26.8	67.7	15.3	24
**D18-Cl:L8-BO**	**Tol**	**DTT**	**0.90**	**26.37**	**76.3**	**18.03**	**This work**
**D18-Cl:L8-BO-X**	**Tol**	**DTT**	**0.89**	**26.78**	**79.6**	**19.04**	**This work**

Supplementary Table 14. Representative SD devices from literature.

D/A	Solvent	Additive	Annealing	V_{OC} (V)	J_{SC} (mA cm^{-2})	FF (%)	PCE (%)	**Ref**
**D18-Cl/L8-BO-X**	**Toluene**	**None**	**None**	**0.893**	**26.78**	**79.6**	**19.04**	**This**
**D18-Cl/L8-BO**	**Toluene**	**None**	**None**	**0.896**	**26.37**	**76.3**	**18.03**	**work**
PM6/T8	CF	CN	Thermal	0.864	26.98	77.13	17.98	25
PM6/BTP-eC9:PC71BM:PAEF	CF	DIO	Thermal	0.857	27.74	78.21	18.59	26
PM6/PYF-T-o	Toluene	CN	Thermal	0.905	25.16	69.5	15.82	27
PM6/BTP-eC9	CF	None	Thermal	0.86	26.68	74.6	17.13	28
PM6/L8-BO	CB/CF	WAX	None	0.89	26.11	80.6	18.74	29
PM6/PM7/Y6:BO-4Cl	CB/CF	DTBF	Thermal	0.849	26.3	77.9	17.4	30
PNTB6-Cl/N3	CB/CF	DIO	None	0.857	26.58	77.26	17.59	31
PM6/BO-4Cl:BTP-S2	CF	DIO	Thermal	0.861	27.14	78.04	18.16	32
PM6:DRTB-TC4/BTP-eC9:IDIC	CF	DIO	Thermal	0.850	26.43	77.25	17.35	33
PM6/Y6	CF	None	None	0.834	25.90	75.68	16.35	34
PM6/Y6:FBR	CB/CF	CN	Thermal	0.83	26.3	75.6	16.4	35
PM6/Y6	CB/CF	DIO	Thermal	0.800	24.5	73.5	14.42	36
PTQ10/IDIC	CF	None	Thermal	0.943	18.75	69.7	12.32	37
PM6/BO-4Cl:L8-BO	o-xylene	DIO	Thermal	0.842	27.14	78.28	17.89	38
PM6/Y6	CB/CF	CN	Thermal	0.87	24.3	69.1	14.50	39
PM6/Y6	CB/CF	None	Thermal	0.80	25.28	71.3	14.42	40
PBDB-T/ThPF-4F	o-DCB/CB	CN	Thermal	0.80	24.18	61.89	11.97	41
ZnP-TSEH/4TIC:6TIC	CF/DCM	GOMe	Thermal	0.844	26.33	77.31	17.18	42
PTAP1/Y6-BO	CB/CF	DIO	Thermal	0.844	26.78	75.9	17.14	43
PM6/Y6	CF	None	Thermal	9.90	1.93	70.53	13.47	44
PBTz-TC/IT-4F	TMB	None	None	0.84	20.91	72.69	12.81	45
PM6:PM7/Y6:O1-2F	CF	CN	Thermal	0.866	26.97	78.1	18.23	46
PM6/ BTP-eC9	CB/CF	P-Cl	Thermal	0.853	27.81	80.50	19.10	47
PM6/BTP-BO-4Cl	o-DCB	None	Thermal	0.850	24.07	64.0	13.09	46
PM6/PYF-T-o	CB/CF	CN	Thermal	0.90	23.86	71.17	15.28	48
PBDB-T/ PYT	CF	CN	Thermal	0.891	23.03	73.98	15.17	49
PM6/Y6:PC71BM	CB/CF	DIO	None	0.83	26.6	77.1	17.0	50
PM6/PYT	CB/CF	CN	Thermal	0.91	23.07	77.0	16.05	51
PM6/Y7:BTA-UD-4F	o-xylene	DIO	Thermal	0.85	27.4	75.18	17.55	52

PM6/L8-BO	CF	DIO	Thermal	0.883	26.61	80.39	18.86	[53]
FTAZ/IT-M	Limonene/2-MeTHF	None	Thermal	0.962	18.6	70.07	12.5	[54]
PM6/IT-4F	TMB	None	Thermal	0.84	20.5	75	12.9	[55]
PBDB-T/NCBDT	CF/DCM	None	None	0.824	19.45	62.9	10.04	[56]
PBDB-T/NCBDT	CF/DCM	None	None	0.839	18.39	61.5	9.49	[57]
PBDB-T:ITIC/IDIC	CF/DCB	DIO	Thermal	0.91	16.4	72.8	11.0	[58]
PBDB-TFS1/IT-4F	CB/THF	oDCB	Thermal	0.90	20.3	71	13.0	[59]
D18/T9TBO-F:Y6-O	CB/CF	None	None	0.87	27.90	78.81	19.13	[60]
PM6/PY-V-γ	o-XY	CN	Thermal	0.913	24.9	77.7	17.7	[61]
D18/PY-FT	CB/Toluene	CN	Thermal	0.925	24.2	76.1	17.0	[62]
D18/BS3TSe-4F:Y6-O	CB/CF	None	None	0.845	29.41	76.56	19.03	[63]
PM6/Y6-BO	CF	CN	Thermal	0.847	26.2	77.5	17.2	[64]
PBDB-T:IT-M	CB	DIO	None	0.751	24.66	63.57	11.91	[65]
PTB7-Th/IEICO-4F	o-XY/n-butanol	CN	None	0.69	25.4	68.0	12.0	[66]
PTB7-Th/FOIC:N2200	CB	DIO	None	0.72	24.17	68.6	12.27	[67]
PTB7-Th/p-DTS(FBTTH2)2	o-XY	DIO	None	0.73	20.82	69.27	10.14	[68]
D18/ /N3	CB/ n-octane/CF	None	CF-SVA	0.834	27.79	75.61	17.52	[69]
PM6/L15	CB/CF	CN	Thermal	0.94	23.58	73.17	16.15	[70]
PT2/Y6	CB/CF	DIO	Thermal	0.83	26.7	74.4	16.5	[71]
PM6/IT-4F:F8IC	CB	None	Thermal	0.79	25.6	69.8	14.2	[72]
PM6/PY-IT:PDI-2T	CF	CN	None	0.949	23.97	70.48	16.03	[73]
D18/L8-BO	CB/CF	None	Thermal	0.918	26.86	77.25	19.05	[74]
PM6/L8-BO	o-XY	None	None	0.867	25.37	77.23	17.07	[75]
PTB7-Th/FOIC1	CB/o-DCB:2-MeTHF	None	None	0.699	23.8	72.2	12.0	[76]
PM6/BTP-eC9	o-XY	DIO	Thermal	0.840	26.65	0.781	17.48	[77]
D18/BTP-eC11	CF/Tol	None	CF-SVA	0.85	27.6	75.2	17.7	[78]
P2F-EHp/M4-4F	CF/DCM	DBE	Thermal	0.83	25.56	67.14	14.2	[79]
PM6/Y6	CF	DDO:CN	Thermal	0.85	25.51	77.45	16.93	[80]
PTB7-Th/IEICO-4F	CB	CN	Thermal	0.7	24.22	0.64	11.04	[81]

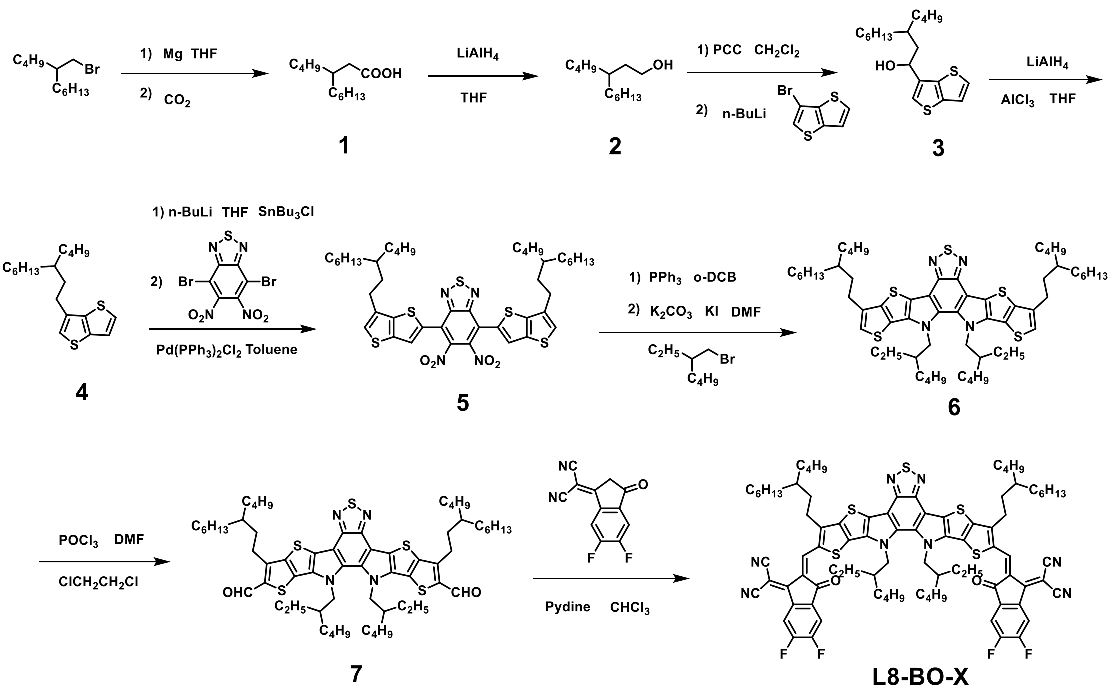

Supplementary Figure 23. The synthetic route to non-fullerene acceptor L8-BO-X.

Starting from commercially available 1-bromo-2-butyloctane, 3-butylnonanoic acid (**compound 1**) was synthesized from 1-bromo-2-butyloctane via the formation of Grignard reagent (2-butyloctyl)magnesium bromide followed by treatment of dry ice. The compound 1 was reacted with $LiAlH_4$ via the reduction reaction to afford 3-butylnonan-1-ol (**compound 2**). This compound 2 was then reacted with PCC afforded 3-butylnonanal, followed by nucleophilic substitution reaction to obtain 3-butyl-1-(thieno[3,2-b]thiophen-3-yl)nonan-1-ol (**compound 3**). 3-(3-butylnonyl) thieno[3,2-b] thiophene (**compound 4**) was prepared by the dihydroxylation of compound 3 by using $LiAlH_4$ and $AlCl_3$. Treatment of compound 4 with n-BuLi followed by quenching with $SnBu_3Cl$ afforded tributyl(6-(3-butylnonyl)thieno[3,2-b]thiophen-2-yl)stannane, which was subjected to Stille cross-coupling reaction with 4,7-dibromo-5,6-dinitrobenzo[c][1,2,5]thiadiazole to furnish 4,7-bis(6-(3-butylnonyl)thieno[3,2-b]thiophen-2-yl)-5,6-dinitrobenzo[c][1,2,5]thiadiazole (**compound 5**). The compound

5 was then converted to 3,9-bis(3-butylnonyl)-12-(2-ethylbutyl)-13-(2-ethylhexyl)-12,13-dihydro-[1,2,5]thiadiazolo[3,4-e]thieno[2'',3'':4',5']thieno[2',3':4,5]pyrrolo[3,2-g]thieno[2',3':4,5]thieno[3,2-b]indole (**compound 6**) through the double intramolecular Cadogan reductive cyclization of compound 5 with PPh_3, followed by the reaction with 1-bromo-2-ethylhexane under alkaline condition. The Vilsmeier-Haak reaction of compound 6 with $POCl_3$ and DMF resulted in 3,9-bis(3-butylnonyl)-12,13-bis(2-ethylhexyl)-12,13-dihydro-[1,2,5]thiadiazolo[3,4-e]thieno[2'',3'':4',5']thieno[2',3':4,5] pyrrolo[3,2-g]thieno[2',3':4,5]thieno[3,2-b]indole-2,10-dicarbaldehyde (**compound 7**). This compound 7 was further converted to the finally desired product 2,2'-((2Z,2'Z)-((3,9-bis(3-butylnonyl)-12,13-bis(2-ethylhexyl)-12,13-dihydro-[1,2,5]thiadiazolo[3,4-e]thieno[2'',3'':4',5']thieno[2',3':4,5]pyrrolo[3,2-g]thieno[2',3':4,5]thieno[3,2-b]indole-2,10-diyl)bis(methanylylidene))bis(5,6-difluoro-3-oxo-2,3-dihydro-1H-indene-2,1-diylidene))dimalononitrile (**L8-BO-X**) via the Knoevenagel condensation reaction of compound 7 with 2-(5,6-difluoro-3-oxo-2,3-dihydro-1H-inden-1-ylidene)malononitri-le. The NMR spectra and mass spectra of the corresponding intermediates and final product L8-BO-X acceptor are provided in Supplementary Figs.24-33.

Synthesis of 3-butylnonan-1-ol (compound 2).

To an oven-dried 25 mL two-necked round-bottomed flask was added magnesium turnings (1.27 g, 52.96 mmol), three grains of iodine and anhydrous THF (10 mL) under nitrogen atmosphere. The reaction mixture was heated to reflux, and then the 5-(bromomethyl)undecane (12 g, 48.15 mmol) dissolved in anhydrous THF (10 mL) was added dropwise at such a rate that the reaction continued to reflux without additional heating. After completion, the reaction mixture was refluxed for another two hours at

80 °C. The reaction mixture was then allowed to warm to room temperature, and the prepared Grignard reagent was transferred to an oven-dried 250 mL two necked round-bottomed flask containing anhydrous THF (80 mL). To this mixture was added dry ice at -78 °C under nitrogen atmosphere. After stirring at room temperature for 3 hours, the mixture was poured into water and the PH of the solution was turned to 1-2 by adding concentrated hydrochloric acid. Afterwards, the mixture was extracted with ethyl acetate, dried over anhydrous $MgSO_4$, filtered and then removed solvent under reduced pressure to afford compound **1**, which is used directly in the next reaction step. To an oven-dried 250 mL two-necked round-bottomed flask was added anhydrous THF (80 mL). Under nitrogen atmosphere, $LiAlH_4$ (7.31 g, 192.6 mmol) was added slowly in several batches to the flask at 0 °C. After completion, the mixture was warmed to room temperature and stirred overnight. Subsequently, the mixture was cooled to 0 °C, and the obtained compound **1** dissolved in anhydrous THF (20 mL) was added dropwise. After the addition, the reaction mixture was heated to 65 °C and stirred for 6 hours. After cooling to room temperature, the mixture was cautiously poured into ice and the PH of the solution was turned to 1-2 by adding concentrated hydrochloric acid. The mixture was then extracted with ethyl acetate, dried over anhydrous $MgSO_4$, filtered and concentrated. The residue was purified by column chromatography on silica gel with hexane: ethyl acetate (6:1, v:v) as an eluent to yield product as a yellowish brown liquid. (4.3 g, 44.5%). ^{1}H NMR (400 MHz, $CDCl_3$, δ): 3.68-3.64 (t, 2H), 1.54-1.50 (q, 2H), 1.41 (m, 1H), 1.25 (m, 16H), 0.91-0.86 (m, 6H).

Synthesis of 3-butyl-1-(thieno[3,2-b]thiophen-3-yl)nonan-1-ol (compound 3).

To a 250 mL flask was added compound **2** (4.3 g, 21.49 mmol) and dichloromethane (120 mL). Then the pyridinium chlorochromate (PCC) (6.95 g, 32.24 mmol) was added at room temperature. After stirring at room temperature for 3 hours, the mixture was filtered and the filtrate was concentrated under reduced pressure to afford crude product, which was purified by column chromatography on silica gel with hexane: dichloromethane (1:1.5) as an eluent to yield 3-butylnonanal. To an oven-dried 250 mL

two-necked round-bottomed flask was added 3-bromothieno[3,2-b]thiophene (2.2 g, 10 mmol) and anhydrous Et_2O (60 mL). n-BuLi (4 mL, 10 mmol, 2.5 M in hexane) was added dropwise at -78 °C under nitrogen atmosphere. After stirring at -78 °C for another 1.5 hours, the freshly prepared 3-butylnonanal (3.97 g, 20 mmol) was added quickly. The mixture was then allowed to warm to room temperature and stirred overnight. The mixture was poured into water, followed by extraction with dichloromethane. The combined extracts was concentrated and the residue was purified by column chromatography on silica gel with hexane: ethyl acetate (20:1, v:v) as an eluent to give the product as a colorless liquid. (2.1 g, 62%). ^{1}H NMR (400 MHz, $CDCl_3$, δ): 7.41-7.39 (q, 1H), 7.25 (s, 1H), 7.21-7.20 (d, 1H), 4.99-4.95 (q, 1H), 1.87-1.82 (m, 3H), 1.51 (m, 1H), 1.27 (m, 16H), 0.90-0.85 (m, 6H).

Synthesis of 3-(3-butylnonyl)thieno[3,2-b]thiophene (compound 4).

To an oven-dried 250 ml three-necked round-bottomed flask was added anhydrous Et_2O (60 mL). Under nitrogen stream, $AlCl_3$ (1.24 g, 9.30 mmol) and $LiAlH_4$ (0.71 g, 18.61 mmol) were added slowly in several batches at 0 °C. After the addition, the mixture was warmed to room temperature and stirred overnight. The mixture was further cooled to 0 °C, and the compound **3** (2.1 g, 6.20 mmol) dissolved in anhydrous Et_2O (10 mL) was added dropwise. After stirring at 40 °C for 6 hours, the mixture was cooled to room temperature, then cautiously poured into ice. The PH of the solution was turned to 1-2 by adding the concentrated hydrochloric acid. The organic phase was separated and extracted with dichloromethane. The combined extracts were dried over anhydrous $MgSO_4$, filtered and concentrated to give crude product, which was further purified by column chromatography on silica gel with hexane as an eluent to get the product as a colorless liquid. (1.9 g, 95%). ^{1}H NMR (400 MHz, $CDCl_3$, δ): 7.37-7.35 (q, 1H), 7.25 (s, 1H), 7.24-6.99 (d, 1H), 2.73-2.68 (t, 2H), 1.74-1.68 (m, 2H), 1.42 (m, 1H), 1.29-1.27 (m, 16H), 0.92-0.87 (m, 6H).

Synthesis of 4,7-bis(6-(3-butylnonyl)thieno[3,2-b]thiophen-2-yl)-5,6-dinitrobenzo[c]

[1,2,5]thiadiazole (compound 5).

To an oven-dried 100 ml two-necked round-bottomed flask was added compound **4** (1.9 g, 5.89 mmol) and anhydrous THF (60 mL). n-BuLi (2.47 mL, 6.18 mmol, 2.5 M in hexane) was added dropwise at -78 °C under nitrogen atmosphere. After stirring at -78 °C for 1.5 hours, Tri-n-butyltin chloride (2.88 g, 8.84 mmol) was added. The mixture was allowed to warm to room temperature and stirred overnight. The reaction mixture was quenched by water, extracted with dichloromethane, dried over anhydrous $MgSO_4$, filtered and concentrated under reduced pressure to give crude tributylstannane-substituted derivative, which was used without further purification. The crude tributylstannane-substituted derivative was transferred to a 100 ml two-necked round-bottomed flask containing 4,7-dibromo-5,6-dinitrobenzo[c][1,2,5]thiadiazole (0.942 g, 2.45 mmol), $Pd(PPh_3)Cl_2$ (86 mg, 0.123 mmol) and toluene (50 mL). Under nitrogen atmosphere, the reaction mixture was heated to reflux overnight. After cooling to room temperature, the mixture was extracted with dichloromethane, the extracts were concentrated under reduced pressure and the residue was further purified by column chromatography on silica gel with hexane: dichloromethane (4:1, v:v) as an eluent to afford the product as a red solid. (1.5 g, 70.5%). ^{1}H NMR (400 MHz, $CDCl_3$, δ): 7.71 (s, 2H), 7.18 (s, 2H), 2.78-2.74 (t, 4H), 1.78-1.72 (m, 4H), 1.42-1.41 (m, 2H), 1.30-1.29 (m, 32 H), 0.93-0.87 (m, 16H).

Synthesis of 3,9-bis(3-butylnonyl)-12,13-bis(2-ethylhexyl)-12,13-dihydro-[1,2,5]thiadiazolo[3,4-e]thieno[2'',3'':4',5']thieno[2',3':4,5]pyrrolo[3,2-g]thieno[2',3':4,5]thieno[3,2-b]indole (compound 6).

To a 250 ml round-bottomed flask was added compound **5** (0.64 g, 0.74 mmol), triphenylphosphine (1.94 g, 7.4 mmol) and anhydrous 1,2-dichlorobenzene (*o*-DCB, 10 mL) under nitrogen atmosphere. The reaction mixture was heated to 180 °C and stirred overnight. After cooling to room temperature, methanol was added and the mixture was filtered under reduced pressure to yield crude product. Subsequently, the crude product was transferred to 100 mL two-necked round-bottomed flask containing K_2CO_3 (1.63

g, 11.81 mmol), KI (1.96 g, 11.81 mmol), 1-bromo-2-ethylhexane (2.28 g, 11.81 mmol) and anhydrous DMF (10 mL). The reaction mixture was heated to 80 °C and stirred overnight under nitrogen atmosphere. After cooling to room temperature, the mixture was extracted with dichloromethane and washed with water for three times. The combined organic phase was concentrated under reduced pressure, and the residue was purified by column chromatography on silica gel with hexane: dichloromethane (6:1, v:v) as an eluent to afford an orange yellow solid. (368 mg, 48.6% in two steps). ^{1}H NMR (400 MHz, $CDCl_3$, δ): 7.02 (s, 2H), 4.61-4.58 (m, 4H), 2.83-2.79 (m, 4H), 2.05-2.02 (t, 2H), 1.84-1.79 (m, 4H), 1.46-1.44 (m, 2H), 1.30 (m, 32H), 1.07-1.06 (m, 4H), 0.92-0.88 (m, 24H), 0.62-0.60 (t, 12H).

Synthesis of 3,9-bis(3-butylnonyl)-12,13-bis(2-ethylhexyl)-12,13-dihydro-[1,2,5] thiadiazolo[3,4-e]thieno[2'',3'':4',5']thieno[2',3':4,5]pyrrolo[3,2-g]thieno[2', 3':4,5]thieno[3,2-b]indole-2,10-dicarbaldehyde (compound 7).

To a solution of anhydrous DMF (4 mL) was added $POCl_3$ (1 mL) dropwise at 0 °C under nitrogen atmosphere. After the completion, the reaction mixture was stirred at room temperature for another 30 mins, which is to prepare Vilsmeier reagent. The freshly prepared Vilsmeier reagent was then added dropwise to a solution of compound **6** (368 mg, 0.358 mmol) and anhydrous 1,2-dichloroethane (20 mL) at room temperature. The reaction mixture was heated to 80 °C and stirred overnight under nitrogen atmosphere. After cooling to room temperature, the mixture was quenched by saturated Na_2CO_3 and stirred for another 30 mins. The mixture was extracted with dichloromethane, and washed with water for three times. The combined extracts were concentrated under reduced pressure and the residue was purified by column chromatography on silica gel with hexane: dichloromethane (1:1, v:v) to give the product as a bright yellow solid. (0.32 g, 83.2%). ^{1}H NMR (400 MHz, $CDCl_3$, δ): 10.15 (s, 2H), 4.64-4.61 (m, 4H), 3.19-3.15 (t, 4H), 1.99 (m, 2H), 1.88-1.86 (m, 4H), 1.50-1.49 (m, 2H), 1.33-1.25 (m, 32H), 1.06-1.04 (m, 4H), 0.93-0.90 (m, 24H), 0.66-0.58 (m, 12H). MS (MALDI-TOF) *m/z*: $[M + H]^{+}$calcd for $C_{62}H_{90}N_4O_2S_5$, 1082.57, found:

1083.57.

Synthesis of 2,2'-((2Z,2'Z)-((3,9-bis(3-butylnonyl)-12,13-bis(2-ethylhexyl)-12,13-dihydro-[1,2,5]thiadiazolo[3,4-e]thieno[2'',3'':4',5']thieno[2',3':4,5]pyrrolo[3,2-g]thieno[2',3':4,5]thieno[3,2-b]indole-2,10-diyl)bis(methanylylidene))bis(5,6-difluoro-3-oxo-2,3-dihydro-1H-indene-2,1-diylidene))dimalononitrile (compound L8-BO-X). Under nitrogen atmosphere, compound **7** (80 mg, 0.0738 mmol), 2-(5,6-difluoro-3-oxo-2,3-dihydro-1H-inden-1-ylidene)malononitrile (68 mg, 0.295 mmol) and chloroform (30 mL) was added to a 50 mL two-necked round-bottomed flask. After stirring for 5 minus, pyridine (1 mL) was added. The reaction mixture was heated to 65 °C and stirred overnight. After removal of solvent of reaction mixture under reduced pressure, the residue was purified by column chromatography on silica gel with hexane: dichloromethane (1:1, v:v) as an eluent to afford the product as a dark solid. (92 mg, 82.6%). ^{1}H NMR (400 MHz, $CDCl_3$, δ): 9.09 (s, 2H), 8.52-8.48 (q, 2H), 7.72-7.68 (t, 2H), 4.80 (m, 4H), 3.21-3.17 (t, 4H), 2.13-2.10 (m, 2H), 1.80-1.78 (m, 4H), 1.55-1.53 (m, 2H), 1.40-1.21 (m, 32H), 1.05-1.04 (m, 6H), 0.93 (m, 12H), 0.90 (m, 12H), 0.77-0.68 (m, 12H). ^{13}C NMR (100 MHz, $CDCl_3$, δ): 185.40, 158.23, 155.05, 154.92, 153.73, 152.50, 152.44, 152.36, 152.30, 146.92, 144.31, 137.07, 136.00, 135.95, 135.41, 134.50, 133.86,133.80, 133.30, 132.46, 130.04, 119.34, 114.29, 114.12,113.88, 112.98, 111.91, 111.73, 68.03, 55.05, 39.79, 37.20, 34.69, 32.70, 32.42, 31.39, 29.18, 29.08, 28.25, 27.14, 27.07, 26.81, 25.98, 22.64, 22.59, 22.51, 22.21, 22.12, 13.59, 13.54, 13.13, 9.59, 9.51. MS (MALDI-TOF) *m*/*z*: $[M + H]^+$calcd for $C_{86}H_{94}F_4N_8O_2S_5$, 1056.60, found: 1057.61.

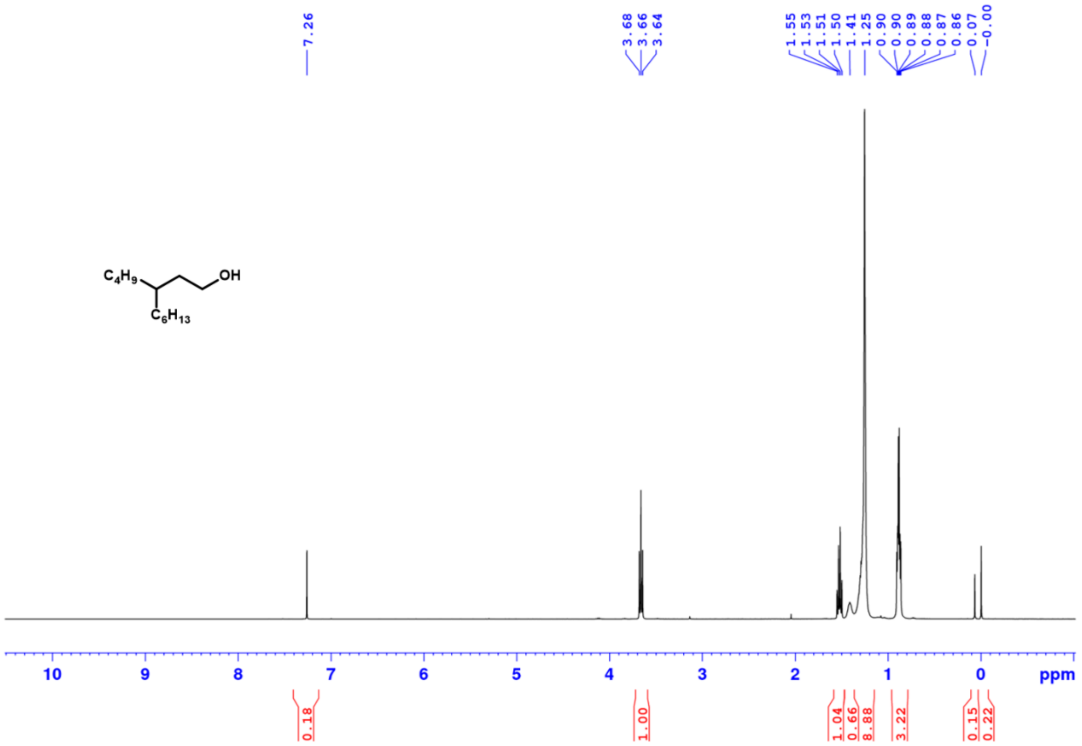

Supplementary Figure **24.** ^{1}H NMR spectrum of compound **2**.

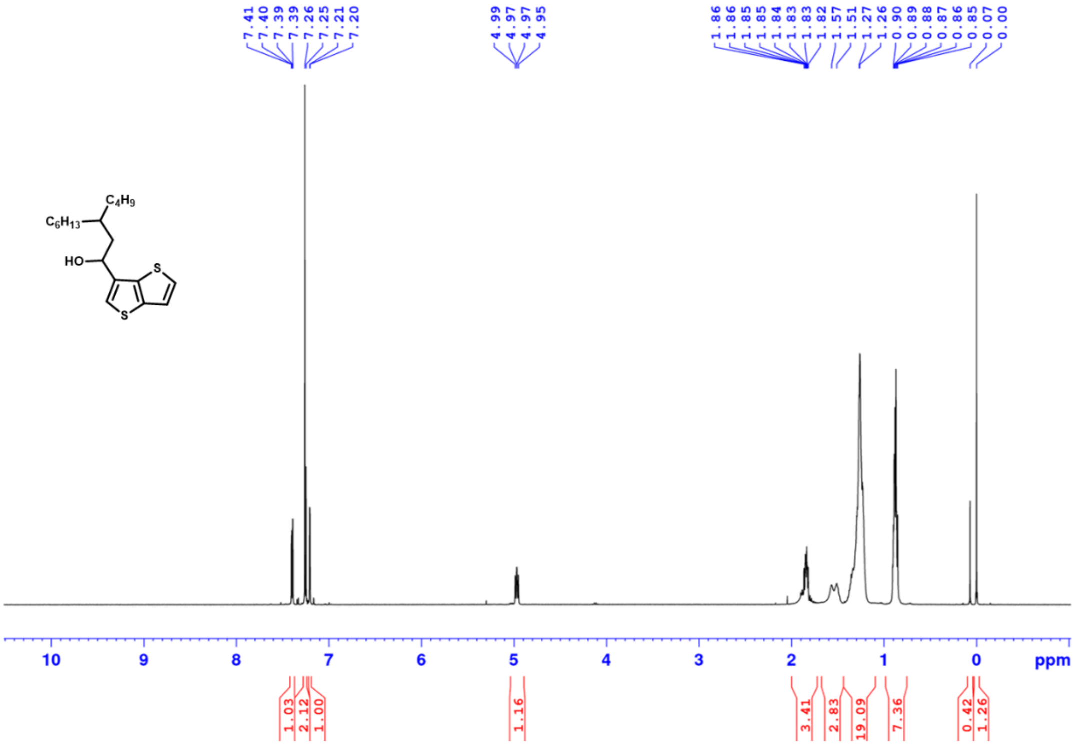

Supplementary Figure 25. ^{1}H NMR spectrum of compound **3**.

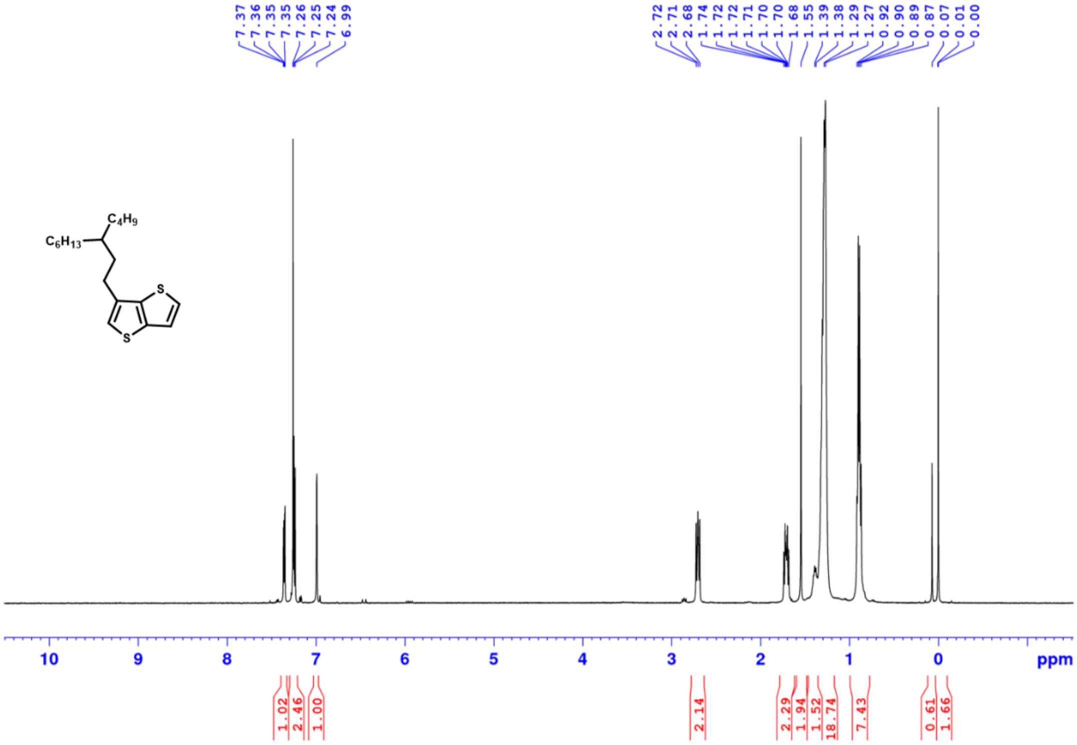

Supplementary Figure 26. ^{1}H NMR spectrum of compound **4**.

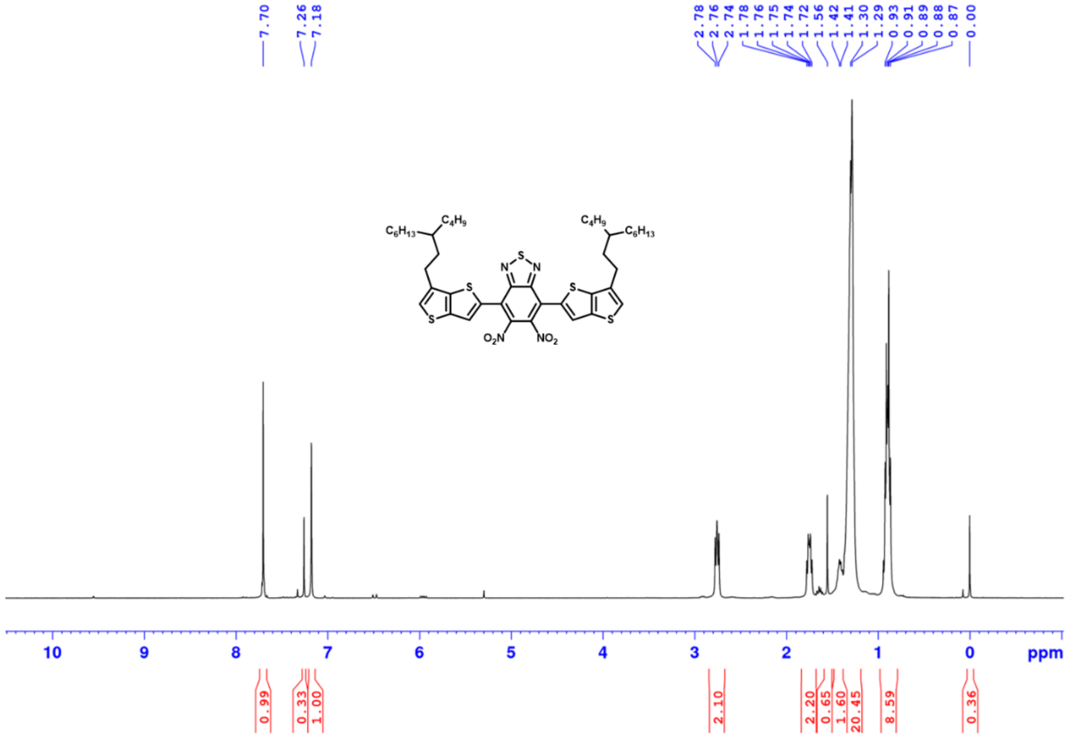

Supplementary Figure 27. ^{1}H NMR spectrum of compound **5**.

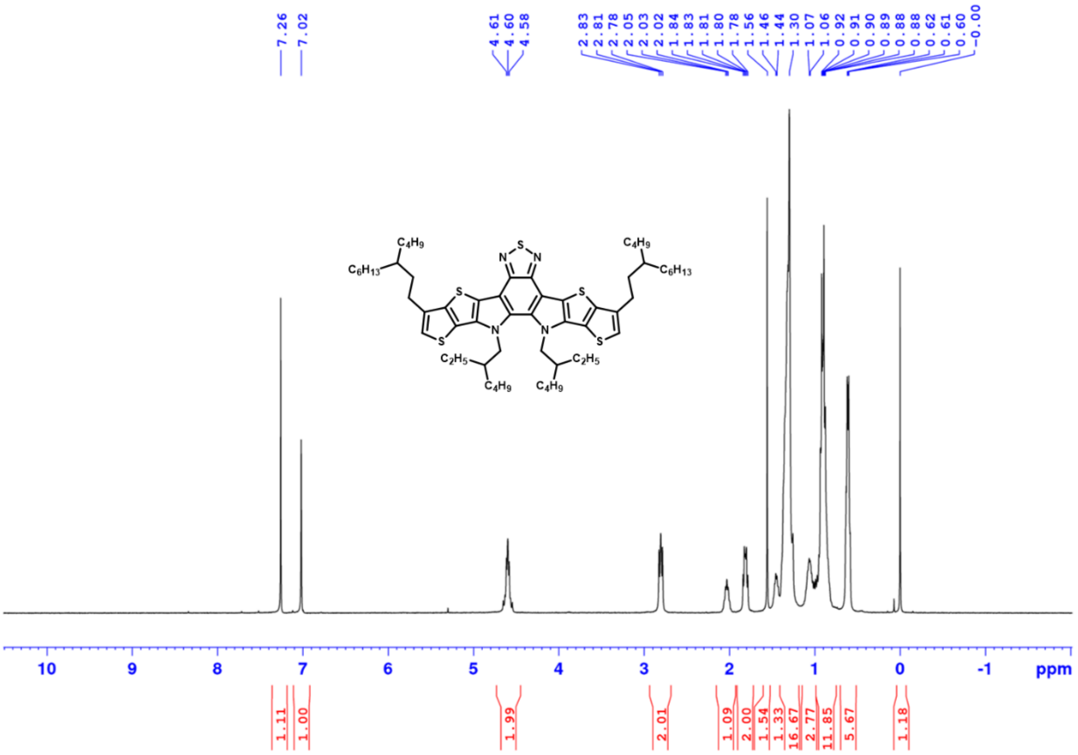

Supplementary Figure 28. ^{1}H NMR spectrum of compound **6**.

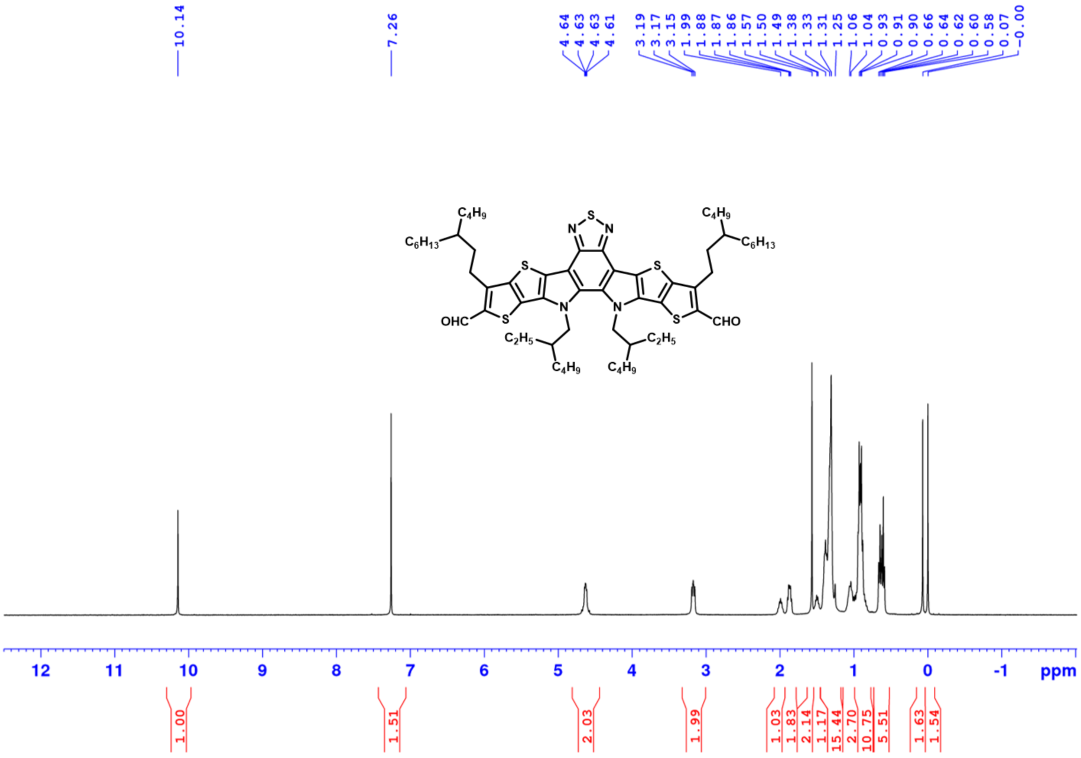

Supplementary Figure 29. ^{1}H NMR spectrum of compound **7**.

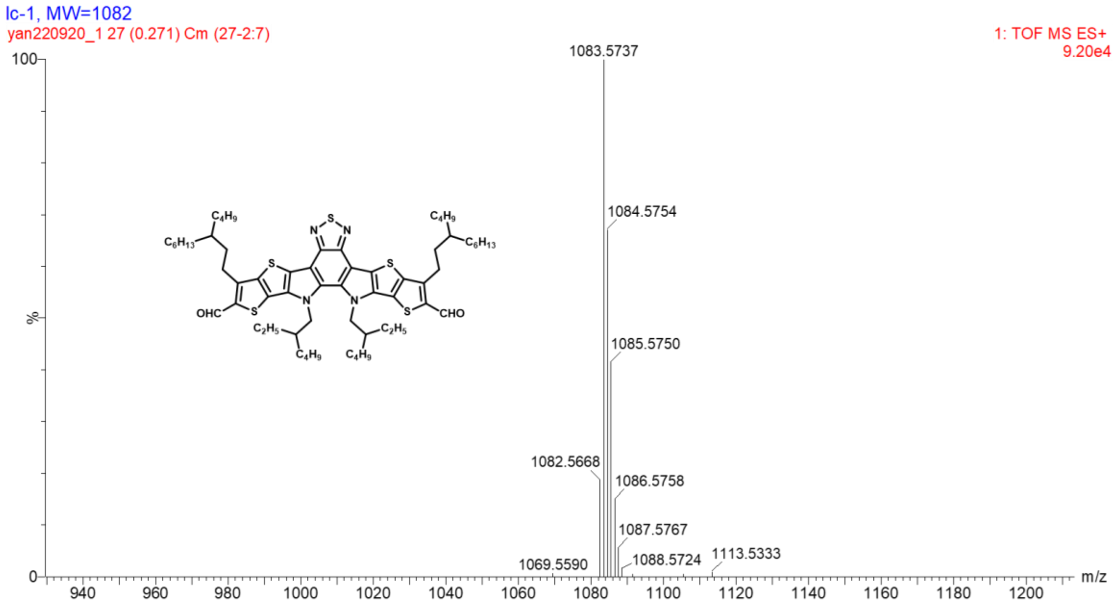

Supplementary Figure 30. MS spectrum (MALDI-TOF) of compound **7**.

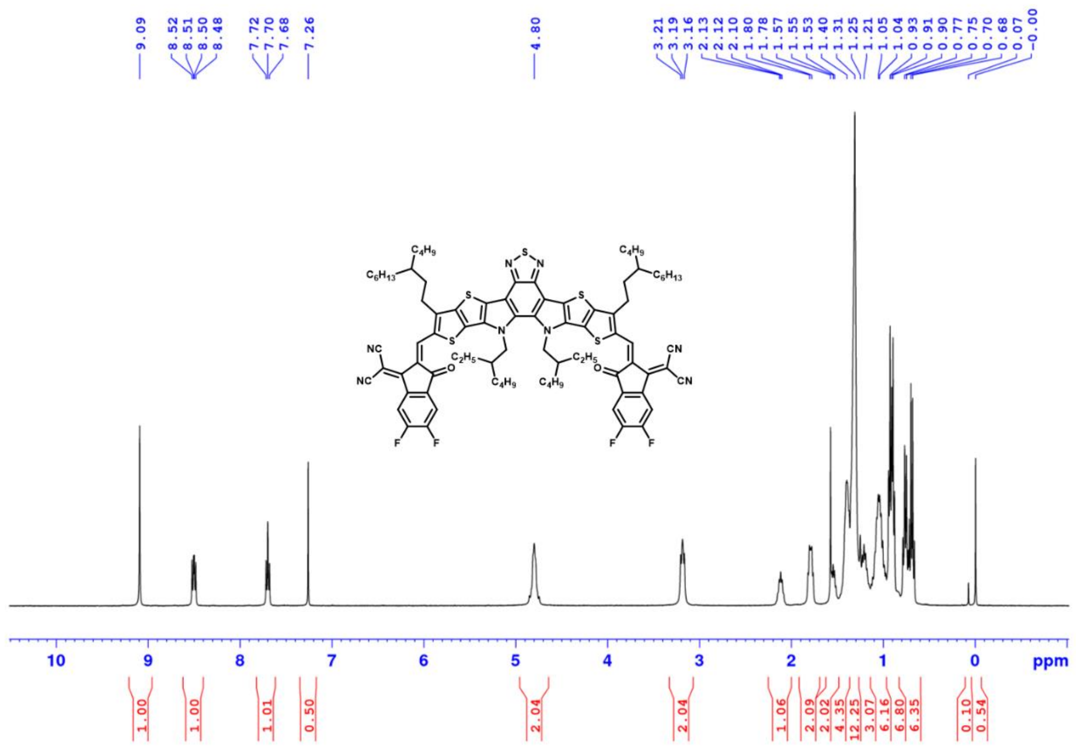

Supplementary Figure 31. ^{1}H NMR spectrum of compound **L8-BO-X**.

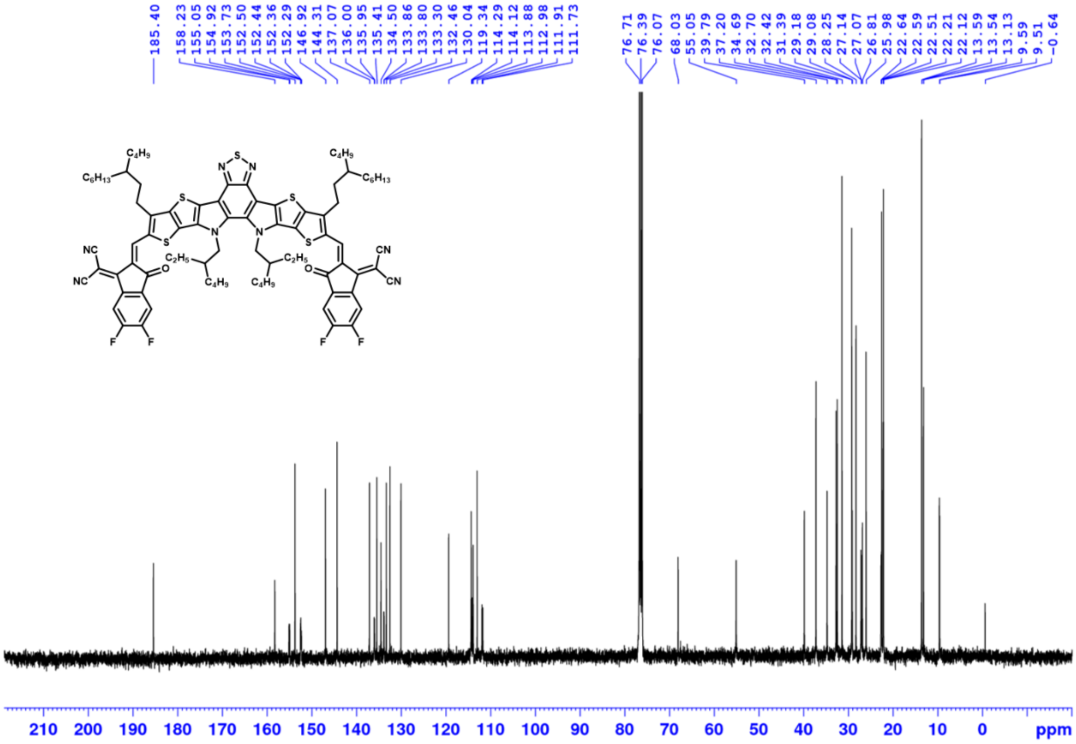

Supplementary Figure 32. ^{13}C NMP spectrum of compound **L8-BO-X**.

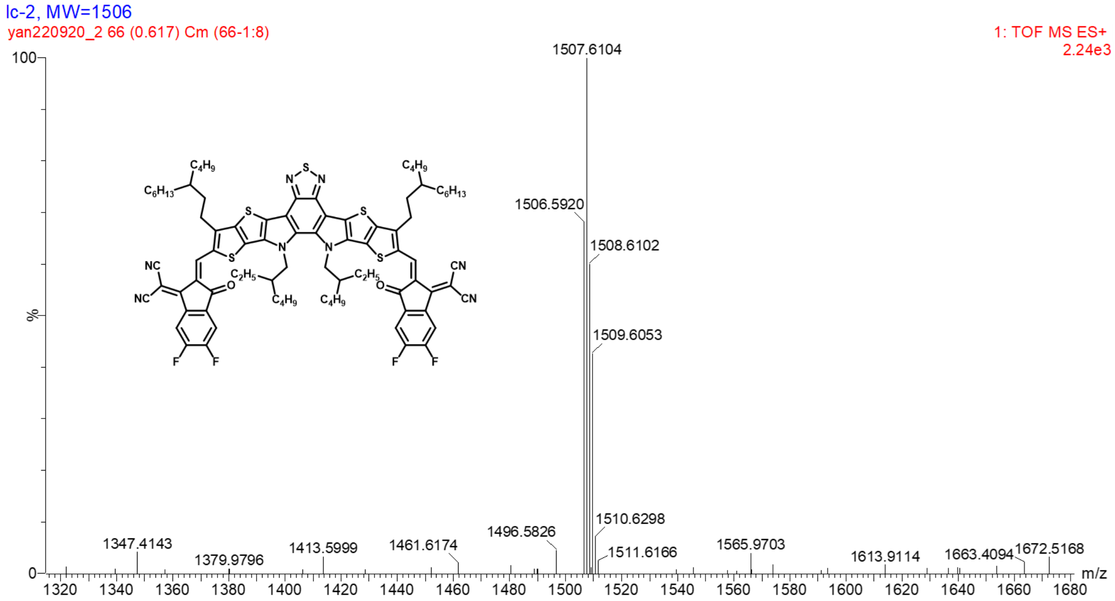

Supplementary Figure 33. MS spectrum (MALDI-TOF) of compound **L8-BO-X**.

Supplementary References

1 Xia, H. *et al.* Novel Oligomer Enables Green Solvent Processed 17.5% Ternary Organic Solar Cells: Synergistic Energy Loss Reduction and Morphology Fine-Tuning. *Adv. Mater.* **34**, e2107659 (2022).

2 Song, X. *et al.* Investigation of tunable halogen-free solvent engineering on aggregation and miscibility towards high-performance organic solar cells. *Nano Energy* **91** (2022).

3 Wang, D. *et al.* High-Performance Organic Solar Cells from Non-Halogenated Solvents. *Adv. Funct. Mater.* **32** (2021).

4 Li, H. *et al.* A general enlarging shear impulse approach to green printing large-area and efficient organic photovoltaics. *Energy Environ. Sci* **15**, 2130-2138 (2022).

5 Liu, H. *et al.* Ternary polymerization strategy to approach 12% efficiency in all-polymer solar cells processed by green solvent and additive. *Chem. Eng. J.* **429** (2022).

6 Wan, J. *et al.* All-Green Solvent-Processed Planar Heterojunction Organic Solar Cells with Outstanding Power Conversion Efficiency of 16%. *Adv. Funct. Mater.* **32** (2021).

7 Zhao, H. *et al.* Kinetics Manipulation Enables High-Performance Thick Ternary Organic Solar Cells via R2R-Compatible Slot-Die Coating. *Adv. Mater.* **34**, e2105114 (2022).

8 Guo, J., Wu, Y., Wang, W., Wang, T. & Min, J. Achieving 12.6% Efficiency in Single-Component Organic Solar Cells Processed from Nonhalogenated Solvents. *Sol. RRL* **6** (2022).

9 Fu, Z. *et al.* Enhancement Efficiency of Organic Photovoltaic Cells via Green Solvents and Nontoxic Halogen-Free Additives. *Adv. Sustainable Syst.* **5** (2021).

10 Chen, H. *et al.* A guest-assisted molecular-organization approach for >17% efficiency organic solar cells using environmentally friendly solvents. *Nat. Energy* **6**, 1045-1053 (2021).

11 Wang, D. *et al.* High-performance and eco-friendly semitransparent organic solar cells for greenhouse applications. *Joule* **5**, 945-957 (2021).

12 Fan, B. *et al.* Enabling High Efficiency of Hydrocarbon-Solvent Processed Organic Solar Cells through Balanced Charge Generation and Non-Radiative Loss. *Adv.*

Energy Mater. **11** (2021).

13 Li, Y. *et al.* Additive and High-Temperature Processing Boost the Photovoltaic Performance of Nonfullerene Organic Solar Cells Fabricated with Blade Coating and Nonhalogenated Solvents. *ACS Appl. Mater. Interfaces* **13**, 10239-10248 (2021).

14 Du, Z. *et al.* Insights into Bulk-Heterojunction Organic Solar Cells Processed from Green Solvent. *Sol. RRL* **5** (2021).

15 Kim, C. *et al.* Green solvent-processed, high-performance organic solar cells achieved by outer side-chain selection of selenophene-incorporated Y-series acceptors. *J. Mater. Chem. A* **9**, 24622-24630 (2021).

16 Yu, H. *et al.* A Difluoro-Monobromo End Group Enables High-Performance Polymer Acceptor and Efficient All-Polymer Solar Cells Processable with Green Solvent under Ambient Condition. *Adv. Funct. Mater.* **31**, 2100791 (2021).

17 Xu, X., Yu, L., Yan, H., Li, R. & Peng, Q. Highly efficient non-fullerene organic solar cells enabled by a delayed processing method using a non-halogenated solvent. *Energy Environ. Sci* **13**, 4381-4388 (2020).

18 Dong, S., Jia, T., Zhang, K., Jing, J. & Huang, F. Single-Component Non-halogen Solvent-Processed High-Performance Organic Solar Cell Module with Efficiency over 14%. *Joule* **4**, 2004-2016 (2020).

19 Zhao, H. *et al.* Hot Hydrocarbon-Solvent Slot-Die Coating Enables High-Efficiency Organic Solar Cells with Temperature-Dependent Aggregation Behavior. *Adv. Mater.* **32**, e2002302 (2020).

20 Liao, C.-Y. *et al.* Processing Strategies for an Organic Photovoltaic Module with over 10% Efficiency. *Joule* **4**, 189-206 (2020).

21 Kumari, T. *et al.* A built-in electric field induced by ferroelectrics increases halogen-free organic solar cell efficiency in various device types. *Nano Energy* **68** (2020).

22 Han, X. *et al.* An Alkoxy-Solubilizing Decacyclic Electron Acceptor for Efficient Ecofriendly As-Cast Blade-Coated Organic Solar Cells. *Sol. RRL* **4** (2020).

23 Cheng, J. *et al.* Investigation of halogen-free solvents towards high-performance additive-free non-fullerene organic solar cells. *Org. Electron.* **85** (2020).

24 Zhang, C.-H. *et al.* Methyl functionalization on conjugated side chains for polymer solar cells processed from non-chlorinated solvents. *J. Mater. Chem. C* **8**, 11532-11539 (2020).

25 Sun, R. *et al.* High-speed sequential deposition of photoactive layers for organic solar cell manufacturing. *Nat. Energy* **7**, 1087-1099 (2022).

26 Han, J. *et al.* Vertical Stratification Engineering of Insulating Poly(aryl ether)s Enables 18.6% Organic Solar Cells with Improved Stability. *ACS Energy Lett.* **7**, 2927-2936 (2022).

27 Zhao, C. *et al.* An improved performance of all polymer solar cells enabled by sequential processing via non-halogenated solvents. *Nano Energy* **104**, 107872 (2022).

28 Yin, Z. *et al.* 17.13% Efficiency and Superior Thermal Stability of Organic Solar Cells Based on a Comb-Shape Active Blend. *Energy Environ. Mater.*, e12443 (2022).

29 Xu, X. *et al.* Polymer Solar Cells with 18.74% Efficiency: From Bulk Heterojunction to Interdigitated Bulk Heterojunction. *Adv. Funct. Mater.* **32** (2021).

30 Yu, R. *et al.* Multi-Functional Solid Additive Induced Favorable Vertical Phase Separation and Ordered Molecular Packing for Highly Efficient Layer-by-Layer Organic Solar Cells. *Small* **17**, e2103497 (2021).

31 Ning, H. *et al.* Manipulating the solubility properties of polymer donors for high-performance layer-by-layer processed organic solar cells. *Energy Environ. Sci* **14**, 5919-5928 (2021).

32 Zhan, L. *et al.* Layer-by-Layer Processed Ternary Organic Photovoltaics with Efficiency over 18. *Adv. Mater.* **33**, e2007231 (2021).

33 Zhang, K. N. *et al.* Reducing Limitations of Aggregation-Induced Photocarrier Trapping for Photovoltaic Stability via Tailoring Intermolecular Electron–Phonon Coupling in Highly Efficient Quaternary Polymer Solar Cells. *Adv. Energy Mater.* **12** (2021).

34 Sun, R. *et al.* A Layer-by-Layer Architecture for Printable Organic Solar Cells Overcoming the Scaling Lag of Module Efficiency. *Joule* **4**, 407-419 (2020).

35 Ren, M. *et al.* High-Performance Ternary Organic Solar Cells with Controllable Morphology via Sequential Layer-by-Layer Deposition. *ACS Appl. Mater. Interfaces* **12**, 13077-13086 (2020).

36 Xie, X. *et al.* Vertical Distribution in Inverted Nonfullerene Polymer Solar Cells by Layer-by-Layer Solution Fabrication Process. *Phys. Status Solidi RRL* **15** (2021).

37 Sun, R. *et al.* A universal layer-by-layer solution-processing approach for efficient non-fullerene organic solar cells. *Energy Environ. Sci* **12**, 384-395 (2019).

38 Li, X. *et al.* High-Performance Layer-by-Layer organic solar cells enabled by Non-Halogenated solvent with 17.89% efficiency. *Chem. Eng. J.* **452** (2023).

39 Karuthedath, S. *et al.* Trace Solvent Additives Enhance Charge Generation in Layer-by-Layer Coated Organic Solar Cells. *Small Struct.* **3** (2022).

40 Yang, Y. *et al.* Layer-by-layer slot-die coated high-efficiency organic solar cells processed using twin boiling point solvents under ambient condition. *Nano Res.* **14**, 4236-4242 (2021).

41 Lin, Z., Du, F., Wang, H., Cao, J. & Tang, W. An unfused-ring acceptor enabling ~12% efficiency for layer-by-layer organic solar cells. *J. Mater. Chem. C* **10**, 10511-10518 (2022).

42 Sun, Y. *et al.* Rational control of sequential morphology evolution and vertical distribution toward 17.18% efficiency all-small-molecule organic solar cells. *Joule* **6**, 2835-2848 (2022).

43 Han, P. *et al.* Ladder-Type Thienoacenaphthopyrazine-Based Molecules: Synthesis, Properties, and Application to Construct High-Performance Polymer for Organic Solar Cells. *CCS Chem.*, 1-14 (2022).

44 Zhang, B. *et al.* Fluid Mechanics Inspired Sequential Blade-Coating for High-Performance Large-Area Organic Solar Modules. *Adv. Funct. Mater.* **32** (2022).

45 Deng, J. *et al.* Layer-by-layer and non-halogenated solvent processing of benzodithiophene-free simple polymer donors for organic solar cells. *Chem. Eng. J.* **443** (2022).

46 Chen, X. *et al.* Balancing the Molecular Aggregation and Vertical Phase Separation in the Polymer: Nonfullerene Blend Films Enables 13.09% Efficiency of Organic Solar Cells with Inkjet-Printed Active Layer. *Adv. Energy Mater.* **12** (2022).

47 Zhou, M. *et al.* 19.10% Efficiency and 80.5% Fill Factor Layer-by-Layer Organic Solar Cells Realized by 4-Bis(2-Thienyl)Pyrrole-2,5-Dione Based Polymer Additives for Inducing Vertical Segregation Morphology. *Adv Mater* **35**, 2208279 (2022).

48 Liu, Z. *et al.* 15.28% efficiency of conventional layer-by-layer all-polymer solar cells superior to bulk heterojunction or inverted cells. *Chem. Eng. J.* **450** (2022).

49 Wu, Q. *et al.* High-Performance All-Polymer Solar Cells with a Pseudo-Bilayer Configuration Enabled by a Stepwise Optimization Strategy. *Adv. Funct. Mater.* **31** (2021).

50 Li, Q. *et al.* Vertical Composition Distribution and Crystallinity Regulations Enable High-Performance Polymer Solar Cells with >17% Efficiency. *ACS Energy Lett.* **5**, 3637-3646 (2020).

51 Zhang, Y. *et al.* Layer-by-layer processed binary all-polymer solar cells with efficiency over 16% enabled by finely optimized morphology. *Nano Energy* **93**, 106858 (2022).

52 Gokulnath, T. *et al.* Highly efficient layer-by-layer deposition solar cells achieved with halogen-free solvents and molecular engineering of non-fullerene acceptors. *Chem. Eng. J.* **448** (2022).

53 He, C. *et al.* Versatile Sequential Casting Processing for Highly Efficient and Stable Binary Organic Photovoltaics. *Adv. Mater.* **34**, e2203379 (2022).

54 Ye, L. *et al.* Sequential Deposition of Organic Films with Eco-Compatible Solvents Improves Performance and Enables Over 12%-Efficiency Nonfullerene Solar Cells. *Adv. Mater.* **31**, e1808153 (2019).

55 Dong, S. *et al.* High-Performance Large-Area Organic Solar Cells Enabled by Sequential Bilayer Processing via Nonhalogenated Solvents. *Adv. Energy Mater.* **9** (2019).

56 Zhang, J. *et al.* Efficient non-fullerene organic solar cells employing sequentially deposited donor–acceptor layers. *J. Mater. Chem. A* **6**, 18225-18233 (2018).

57 Zhang, J. *et al.* Sequentially Deposited versus Conventional Nonfullerene Organic Solar Cells: Interfacial Trap States, Vertical Stratification, and Exciton Dissociation. *Adv. Energy Mater.* **9** (2019).

58 Cheng, P. *et al.* Ternary System with Controlled Structure: A New Strategy toward Efficient Organic Photovoltaics. *Adv Mater* **30**, 1705243 (2018).

59 Cui, Y. *et al.* Toward Efficient Polymer Solar Cells Processed by a Solution-Processed Layer-By-Layer Approach. *Adv Mater* **30**, 1802499 (2018).

60 Jiang, K. *et al.* Suppressed recombination loss in organic photovoltaics adopting a planar–mixed heterojunction architecture. *Nat. Energy* **7**, 1076-1086 (2022).

61 Wang, Y. *et al.* Boosting the Fill Factor through Sequential Deposition and Homo Hydrocarbon Solvent toward Efficient and Stable All-Polymer Solar Cells. *Adv. Energy Mater.* **12** (2022).

62 Fu, H. *et al.* A Top-Down Strategy to Engineer ActiveLayer Morphology for Highly Efficient and Stable All-Polymer Solar Cells. *Adv. Mater.* **34**, e2202608 (2022).

63 Gao, W. *et al.* Achieving 19% Power Conversion Efficiency in Planar-Mixed Heterojunction Organic Solar Cells Using a Pseudosymmetric Electron Acceptor. *Adv. Mater.* **34**, e2202089 (2022).

64 Fu, H. *et al.* A Generally Applicable Approach Using Sequential Deposition to Enable Highly Efficient Organic Solar Cells. *Small Methods* **4**, 2000687 (2020).

65 Wang, Y. *et al.* Achieving Balanced Crystallization Kinetics of Donor and Acceptor

by Sequential-Blade Coated Double Bulk Heterojunction Organic Solar Cells. *Adv. Energy Mater.* **10** (2020).

66 Song, Y. *et al.* Semitransparent Organic Solar Cells Enabled by a Sequentially Deposited Bilayer Structure. *ACS Appl. Mater. Interfaces* **12**, 18473-18481 (2020).

67 Wang, Y. *et al.* Sequential Blade - Coated Acceptor and Donor Enables Simultaneous Enhancement of Efficiency, Stability, and Mechanical Properties for Organic Solar Cells. *Adv. Energy Mater.* **10** (2020).

68 Zhao, Y. *et al.* A Sequential Slot-Die Coated Ternary System Enables Efficient Flexible Organic Solar Cells. *Sol. RRL* **3** (2019).

69 Wei, Y. *et al.* A universal method for constructing high efficiency organic solar cells with stacked structures. *Energy Environ. Sci* **14**, 2314-2321 (2021).

70 Li, B. *et al.* Over 16% efficiency all-polymer solar cells by sequential deposition. *Sci. China: Chem.* **65**, 1157-1163 (2022).

71 Weng, K. *et al.* Optimized active layer morphology toward efficient and polymer batch insensitive organic solar cells. *Nat. Commun.* **11**, 2855 (2020).

72 Wan, J. *et al.* High-Performance Pseudoplanar Heterojunction Ternary Organic Solar Cells with Nonfullerene Alloyed Acceptor. *Adv. Funct. Mater.* **30** (2020).

73 Cui, F. Z. *et al.* Ternary-Assisted Sequential Solution Deposition Enables Efficient All-Polymer Solar Cells with Tailored Vertical-Phase Distribution. *Adv. Funct. Mater.* **32**, 2200478 (2022).

74 Wei, Y. *et al.* Binary Organic Solar Cells Breaking 19% via Manipulating the Vertical Component Distribution. *Adv. Mater.* **34**, e2204718 (2022).

75 Xue, J. *et al.* Nonhalogenated Dual-Slot-Die Processing Enables High-Efficiency Organic Solar Cells. *Adv. Mater.* **34**, e2202659 (2022).

76 Xue, P. *et al.* High-Performance Nonfullerene Organic Solar Cells with Unusual Inverted Structure. *Sol. RRL* **4** (2020).

77 Zhang, Y. *et al.* Graded bulk-heterojunction enables 17% binary organic solar cells via nonhalogenated open air coating. *Nat. Commun.* **12**, 4815 (2021).

78 Li, D. *et al.* Non-fullerene acceptor pre-aggregates enable high efficiency pseudo-bulk heterojunction organic solar cells. *Sci. China: Chem.* **65**, 373-381 (2021).

79 Zhang, D. *et al.* Overcoming incompatibility of donors and acceptors by

constructing planar heterojunction organic solar cells. *Nano Energy* **85**, 105957 (2021).

80 Wang, X. *et al.* High-Efficiency (16.93%) Pseudo-Planar Heterojunction Organic Solar Cells Enabled by Binary Additives Strategy. *Adv. Funct. Mater.* **31** (2021).

81 Chaturvedi, N. *et al.* All Slot-Die Coated Non-Fullerene Organic Solar Cells with PCE 11%. *Adv. Funct. Mater.* **31** (2021).
