# Supplementary material for: Auxiliary sequential deposition enables 19%-efficiency organic solar cells processed from halogen-free solvents
Source: Nat Commun. 2023 Oct 31;14:6964. doi: 10.1038/s41467-023-41978-0 (PMC10618449; doi:10.1038/s41467-023-41978-0)
Supplement: Supplementary file 3 — Solar Cells Reporting Summary [file 41467_2023_41978_MOESM3_ESM.pdf]

Corresponding author(s): Yanming Sun, He Yan

Solar Cells Reporting Summary

Nature Research wishes to improve the reproducibility of the work that we publish. This form is intended for publication with all accepted papers reporting the characterization of photovoltaic devices and provides structure for consistency and transparency in reporting. Some list items might not apply to an individual manuscript, but all fields must be completed for clarity.

For further information on Nature Research policies, including our data availability policy, see Authors & Referees.

Experimental design

Please check: are the following details reported in the manuscript?

1. Dimensions

Item	Yes	No	Response
Area of the tested solar cells	☒	☐	Section "Device fabrication and testing."
Method used to determine the device area	☒	☐	Section "Device fabrication and testing." The area was defined by a shadow mask.

2. Current-voltage characterization

Item	Yes	No	Response
Current density-voltage (J-V) plots in both forward and backward direction	☐	☒	Generally, organic photovoltaic devices do not have forward and backward problems. And we only scan the device in forward direction.
Voltage scan conditions<br>*For instance: scan direction, speed, dwell times*	☒	☐	Section "Device fabrication and testing."
Test environment<br>*For instance: characterization temperature, in air or in glove box*	☒	☐	Our devices were characterized at room temperature (ca. 25 Celsius degree) in air.
Protocol for preconditioning of the device before its characterization	☐	☒	No preconditioning protocol
Stability of the J-V characteristic<br>*Verified with time evolution of the maximum power point or with the photocurrent at maximum power point; see ref. 7 for details.*	☒	☐	We measured the stability with time evolution of the maximum power point

3. Hysteresis or any other unusual behaviour

Item	Yes	No	Response
Description of the unusual behaviour observed during the characterization	☐	☒	No hysteresis or other unusual behavior was observed during the characterization of the solar cells. In general, organic solar cells do not have hysteresis problems.
Related experimental data	☐	☒	No hysteresis or other unusual behaviour was observed during the characterization of the solar cells.

4. Efficiency

Item	Yes	No	Response
External quantum efficiency (EQE) or incident photons to current efficiency (IPCE)	☒	☐	As shown in Fig. 2h and Fig. 2i
A comparison between the integrated response under the standard reference spectrum and the response measure under the simulator	☒	☐	There are less than 5% errors between the integrated response under the standard reference spectrum and the response measure under the simulator. We give details in Table 1.
For tandem solar cells, the bias illumination and bias voltage used for each subcell	☐	☒	We only fabricated single-junction solar cells.

5. Calibration

Item	Yes	No	Response
Light source and reference cell or sensor used for the characterization	☒	☐	Section "Device fabrication and testing."
Confirmation that the reference cell was calibrated and certified	☒	☐	The reference cell 91150-KG5 are certified by Newport

Calculation of spectral mismatch between the reference cell and the devices under test

- [ ] Yes
- [x] No

Explain why this information is not reported/not relevant.

6. Mask/aperture

Size of the mask/aperture used during testing

- [x] Yes
- [ ] No

Section "Device fabrication and testing."

Variation of the measured short-circuit current density with the mask/aperture area

- [ ] Yes
- [x] No

All the current-voltage curves were measured with a single mask.

7. Performance certification

Identity of the independent certification laboratory that confirmed the photovoltaic performance

- [x] Yes
- [ ] No

The device and PCE can be repeated both by HKUST and Beihang University

A copy of any certificate(s)
Provide in Supplementary Information

- [ ] Yes
- [x] No

Explain why this information is not reported/not relevant.

8. Statistics

Number of solar cells tested

- [x] Yes
- [ ] No

The device performance statistics was based on 15 devices.

Statistical analysis of the device performance

- [x] Yes
- [ ] No

Results were included in Table 1.

9. Long-term stability analysis

Type of analysis, bias conditions and environmental conditions
For instance: illumination type, temperature, atmosphere humidity, encapsulation method, preconditioning temperature

- [x] Yes
- [ ] No

See "Device photostability" in the Supplementary Information.
